# Wdpcp, a PCP Protein Required for Ciliogenesis, Regulates Directional Cell Migration and Cell Polarity by Direct Modulation of the Actin Cytoskeleton

**DOI:** 10.1371/journal.pbio.1001720

**Published:** 2013-11-26

**Authors:** Cheng Cui, Bishwanath Chatterjee, Thomas P. Lozito, Zhen Zhang, Richard J. Francis, Hisato Yagi, Lisa M. Swanhart, Subramaniam Sanker, Deanne Francis, Qing Yu, Jovenal T. San Agustin, Chandrakala Puligilla, Tania Chatterjee, Terry Tansey, Xiaoqin Liu, Matthew W. Kelley, Elias T. Spiliotis, Adam V. Kwiatkowski, Rocky Tuan, Gregory J. Pazour, Neil A. Hukriede, Cecilia W. Lo

**Affiliations:** 1 Department of Developmental Biology, University of Pittsburgh School of Medicine, Pittsburgh, Pennsylvania, United States of America; 2 Laboratory of Developmental Biology, National Heart Lung and Blood Institute, National Institutes of Health, Bethesda, Maryland, United States of America; 3 Center for Cellular and Molecular Engineering, Department of Orthopedic Surgery, University of Pittsburgh School of Medicine, Pittsburgh, Pennsylvania, United States of America; 4 Program in Molecular Medicine, University of Massachusetts Medical Center, Worcester, Massachusetts, United States of America; 5 Section on Developmental Neuroscience, National Institute on Deafness and Other Communication Disorders, National Institutes of Health, Bethesda, Maryland, United States of America; 6 Department of Biology, Drexel University, Philadelphia, Pennsylvania, United States of America; 7 Department of Cell Biology, University of Pittsburgh School of Medicine, Pittsburgh, Pennsylvania, United States of America; Stanford University, United States of America

## Abstract

Planar cell polarity (PCP) regulates cell alignment required for collective cell movement during embryonic development. This requires PCP/PCP effector proteins, some of which also play essential roles in ciliogenesis, highlighting the long-standing question of the role of the cilium in PCP. Wdpcp, a PCP effector, was recently shown to regulate both ciliogenesis and collective cell movement, but the underlying mechanism is unknown. Here we show Wdpcp can regulate PCP by direct modulation of the actin cytoskeleton. These studies were made possible by recovery of a *Wdpcp* mutant mouse model. *Wdpcp*-deficient mice exhibit phenotypes reminiscent of Bardet–Biedl/Meckel–Gruber ciliopathy syndromes, including cardiac outflow tract and cochlea defects associated with PCP perturbation. We observed Wdpcp is localized to the transition zone, and in Wdpcp-deficient cells, Sept2, Nphp1, and Mks1 were lost from the transition zone, indicating Wdpcp is required for recruitment of proteins essential for ciliogenesis. Wdpcp is also found in the cytoplasm, where it is localized in the actin cytoskeleton and in focal adhesions. Wdpcp interacts with Sept2 and is colocalized with Sept2 in actin filaments, but in Wdpcp-deficient cells, Sept2 was lost from the actin cytoskeleton, suggesting Wdpcp is required for Sept2 recruitment to actin filaments. Significantly, organization of the actin filaments and focal contacts were markedly changed in Wdpcp-deficient cells. This was associated with decreased membrane ruffling, failure to establish cell polarity, and loss of directional cell migration. These results suggest the PCP defects in *Wdpcp* mutants are not caused by loss of cilia, but by direct disruption of the actin cytoskeleton. Consistent with this, *Wdpcp* mutant cochlea has normal kinocilia and yet exhibits PCP defects. Together, these findings provide the first evidence, to our knowledge, that a PCP component required for ciliogenesis can directly modulate the actin cytoskeleton to regulate cell polarity and directional cell migration.

## Abstract 2

Wdpcp, a protein required for both planar cell polarity and ciliogenesis, regulates cell polarity and alignment via direct modulation of the actin cytoskeleton.

## Author Summary

Cilia are microscopic cell surface hair-like protrusions that can act as antennae to mediate cell signaling. Mutations disrupting ciliogenesis can cause many developmental anomalies associated with syndromes known as “ciliopathies.” Some developmental defects, such as limb polydactyly, arise from disruption of cilia-transduced sonic hedgehog signaling, while other defects, such as aberrant patterning of hair cells in the inner ear, arise from disrupted Wnt signaling resulting in modulation of planar cell polarity (PCP)—a process whereby cells are polarized and aligned. While ciliopathy phenotypes would suggest that cilia are involved in modulating PCP, the mechanistic link between cilia and PCP has been elusive. Our study using a mouse model carrying a mutation in *Wdpcp*, a gene required for both ciliogenesis and PCP, suggest that Wdpcp modulation of PCP involves interactions with the actin cytoskeleton separate from its function in ciliogenesis. We observe Wdpcp localization in cilia, where it is required for recruitment of proteins essential for ciliogenesis. Wdpcp interacts with Sept2, and is also found in actin filaments, where it regulates actin dynamics essential for PCP. Together, these findings show that PCP regulation by Wdpcp is distinct from its function in ciliogenesis and involves direct modulation of the actin cytoskeleton.

## Introduction

The cilium is a microtubule-based organelle projecting from the cell surface with a variety of signaling functions. It plays an important role in development as indicated by the wide spectrum of birth defects associated with ciliopathy syndromes such as Bardet–Biedl (BBS) and Meckel–Gruber (MKS) syndromes [1]. Studies in mice showed some of the developmental anomalies associated with BBS and MKS, such as limb polydactyly, arise from the disruption of Sonic hedgehog (Shh) signaling, a process well described to be cilia transduced [2]–[4]. This entails the regulated trafficking of Shh receptors Patched-1 (Ptch1) [5] and Smoothened (Smo) [6] into the cilia, and the cilium sequestration and processing of Gli transcription factors [7],[8].

Ciliopathy syndromes also exhibit developmental anomalies arising from disruption of Wnt signaling. Noncanonical or β-catenin–independent Wnt signaling specifies planar cell polarity (PCP), a process whereby cells organize and align in a polarized manner relative to the plane of the epithelium. PCP regulated convergent extension cell movement is required for neural tube closure [9]–[11], and patterning of stereocilia polarity in the cochlea requires asymmetric localization of PCP core components [12]–[14]. While the mechanistic link between the cilia and PCP signaling [15]–[17] is not known [18],[19], it is worth noting some PCP components are cilia localized and are required for ciliogenesis [20]–[22]. As cilia disruption can elevate canonical Wnt signaling, the cilium is also proposed to play a role in canonical or β-catenin–dependent Wnt signaling [23],[24]. This may entail maintaining the balance between canonical versus noncanonical Wnt signaling, since cilia disruption can cause opposing effects on canonical versus noncanonical Wnt signaling [24].

In this study, we investigated the role of *Wdpcp* and the cilia in regulating planar cell polarity using a *Wdpcp* mutant mouse model. *Wdpcp* is the homolog of *Drosophila Fritz*, a PCP effector required for patterning hair cell orientation in the *Drosophila* pupal wing [25]. Studies in *Xenopus* embryos [26] showed *Fritz* regulates ciliogenesis and PCP-dependent collective cell movement during gastrulation. However, the role of the cilia and *Wdpcp* (*Fritz*) in the regulation of cell polarity and polarized cell migration was unknown. In this study, we show mice deficient for Wdpcp have phenotypes consistent with MKS/BBS ciliopathy syndromes. This included disruption of ciliogenesis together with developmental anomalies consistent with PCP perturbations. We show Wdpcp is localized in the ciliary transition zone, and also in the actin cytoskeleton and focal adhesions. This involves Wdpcp interactions with Sept2 (septin 2), both in the cilia transition zone and in the actin cytoskeleton. Significantly, the Wdpcp-deficient cells not only exhibited ciliogenesis defect, but also global disruption in the actin cytoskeleton required for maintaining cell polarity and polarized cell migration. To our knowledge, these studies provide the first evidence that a PCP component required for ciliogenesis can regulate planar cell polarity by directly modulating the actin cytoskeleton.

## Results

**Figure 1:**
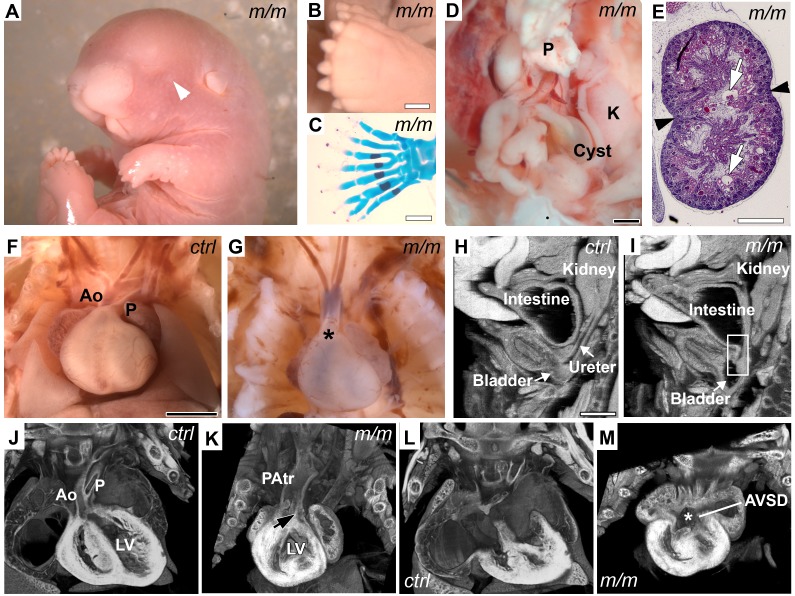
Developmental defects of neonatal *Wdpcp^Cys40^* mutants. (A–I) Necropsy showed *Wdpcp^Cys40^* neonatal mutants have a wide spectrum of defects including facial cleft and anophthalmia (arrowhead in A), polydactyly (B, C), intestinal cysts (D), duplex kidney (black arrowheads in E) with glomerular cysts (white arrows in E), outflow tract defect consisting of a single common trunk (asterisk in G), and cloaca septation defect with abnormal connection between the intestine and bladder (box region in I). (J, K, L, M) Episcopic fluorescence image capture (EFIC) histopathology showed a *Wdpcp^Cys40^* mutant with an incomplete septum unevenly dividing the outflow tract into one large and one small chamber, indicating pulmonary atresia (PAtr; black arrow in K). Also observed was an atrioventricular septal defect (AVSD; asterisk in M). Shown in (J, L) are comparable views of a control heart. Abbreviations: P, pancreas; K, kidney in (D); P, pulmonary trunk in (F) and (J); LV, left ventricle; PAtr, Pulmonary atresia; AVSD, Atrioventricular septal defect. Scale bars, 1 mm in (B, C, D, E, F, H). Magnifications are the same in (F, G, J–M; H, I).

A mutant, named *Wdpcp^Cys40^*, was recovered from an ethylnitrosourea mouse mutagenesis screen exhibiting a wide spectrum of developmental anomalies consistent with MKS/BBS. Particularly notable is their phenotypic similarity to *Mks1* mutant mice that are models of MKS [27],[28]. This included anophthalmia (Figure 1A), central polydactyly (Figure 1B,C), and cysts in the kidney and a variety of other organs (Figure 1D,E). *Wdpcp^Cys40^* mutants also exhibited complex congenital heart defects, usually consisting of persistent truncus arteriosus or pulmonary atresia (Figure 1F,G,J,K), and atrioventricular septal defects (AVSDs; Figure 1L,M). Some mutants had duplex kidney (Figure 1E) and facial clefts and/or cleft palate (Figure 1A). Tracheoesophageal fistula (TEF) due to defects in septation of the oropharynx (unpublished data) and cloacal septation defects were also observed (Figure 1H,I).

**Figure 2:**
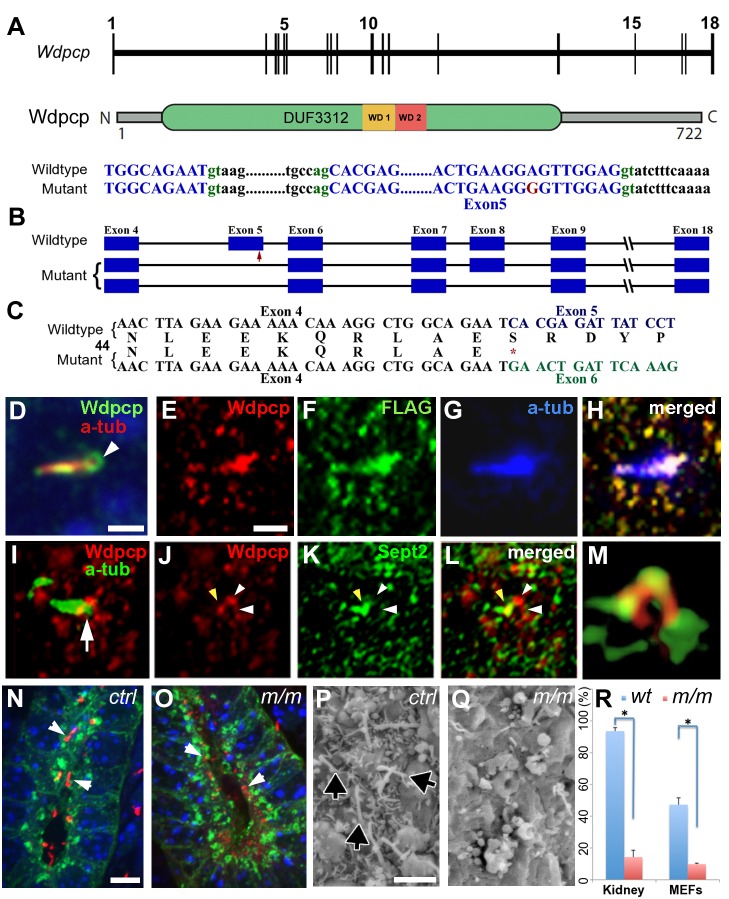
*Wdpcp* mutation, *Wdpcp* cilia localization, and ciliary phenotypes in *Wdpcp^Cys40^* mutants. (A) *Wdpcp* genomic and protein structure. (B) An A>G mutation in *Wdpcp^Cys40^* mutant (red arrow) resulted in two abnormally spliced transcripts—one with exon 5 deleted and one with exons 5 and 8 deleted. (C) Exon 5 deletion generates premature stop, causing protein truncation. (D) IMCD3 cells immunostained with Wdpcp (green) and acetylated α-tubulin (red) antibodies showed Wdpcp localization in the axoneme and ring-like structure (arrowhead) at the cilia base. (E–H) Immunostaining of NIH-3T3 cells transfected with Wdpcp–FLAG showed colocalization of Wdpcp (red) and FLAG (green) in the cilia, with strong staining at the base of the cilium (acetylated α-tubulin, blue). (I–M) Immunostaining of MEFs showed Wdpcp (red, I: Wdpcp) localized in ring-like structure at the base of the cilia (green, I: anti-cetylated α-tubulin). Sept2 (green) showed colocalization with Wdpcp (red, panels L and M). The arrowheads (J–L) point to Wdpcp (J, L) and Sept2 staining (K, L). Note ring-like structure, better seen in 3D isosurface reconstruction (M) of same confocal images as in (J–L). (N, O) Acetylated α-tubulin antibody staining (red) of E15.5 wild-type kidney shows cilia (arrowheads in N), but few cilia were seen in the *Wdpcp^Cys40^* mutant collecting duct (stained green with Dolichos Biflorus Agglutinin; arrowheads in O) and they were much shorter. (P, Q) Scanning EM of E10.5 embryo floor plate showed *Wdpcp^Cys40^* mutant neural epithelium (Q) are less ciliated (see arrow) and lack microvilli as compared to control (P). (R) Percentage of ciliated cells is reduced in *Wdpcp^Cys40^* mutant kidney epithelia and MEFs. Scale bars, 1 µm in (D), 2 µm in (E–L), 5 µm in (N, O), 2 µm in (P, Q).

We mapped the mutation to a 6.36 Mb interval delimited by SNP rs26841005 and rs26856862 on mouse chromosome 11. RT-PCR analysis was carried out using RNA extracted from E12.5 hearts of *Wdpcp^Cys40^* mutant embryos to interrogate transcript expression from the 36 genes in the mapped interval. This analysis revealed an anomalous transcript from *Wdpcp* (NM_145425.3 and NP_663400.2). Further sequencing analysis suggested this was derived from a splicing defect mutation, which was confirmed with genomic DNA sequencing. An A to G substitution was observed at nucleotide 224 of the mRNA (Figure 2A), corresponding to the 8^th^ base before the splice donor site of exon 5 (Figure 2B). As a result, a premature stop codon (S54X) is generated, causing protein truncation after amino acid 54 (Figure 2C). Quantitative PCR analysis with primers covering exons 5–6 showed only low trace amount (0.6%) of normal transcripts, suggesting *Wdpcp^Cys40^* is essentially a null or strong hypomorphic *Wdpcp* mutant allele.

### Wdpcp Required for Recruitment of Proteins for Ciliogenesis

**Figure 3:**
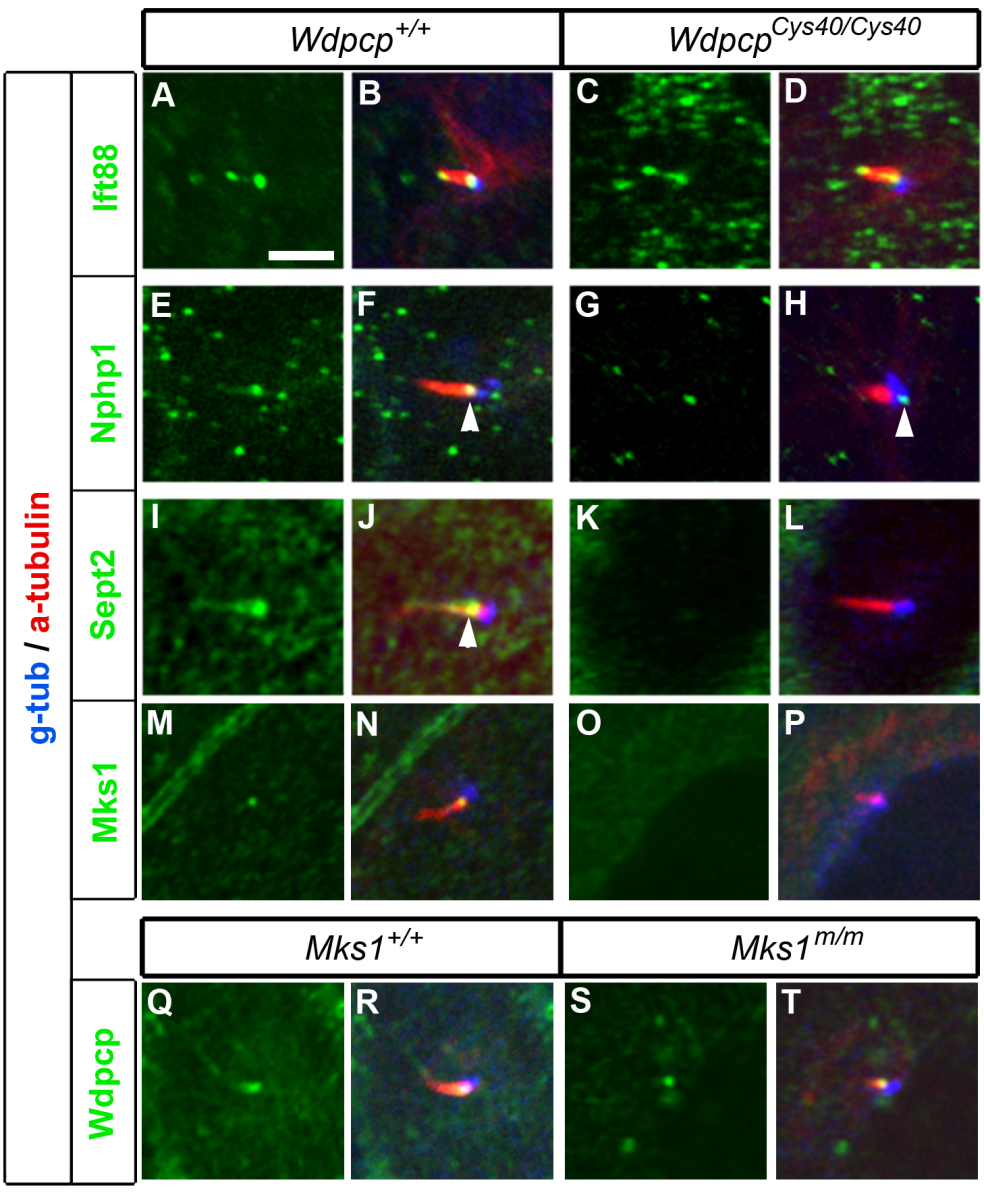
Wdpcp is required for cilia recruitment of Sept2, Mks1, and Nphp1. (A–D) Ift88 is localized correctly to the basal body and ciliary tip in the rare cilium formed in *Wdpcp^Cys40^* mutant MEFs. (E–H) Nphp1 is normally found at the transition zone (E, and arrowhead in F), but in *Wdpcp^Cys40^* mutant MEFs, Nphp1 is mislocalized in the basal body (G, H). (I–L) Sept2 is normally found in the transition zone and sometimes in the ciliary axoneme in control MEFs (I, and arrowhead in J), but is absent from the cilium of *Wdpcp^Cys40^* mutant MEF (K, L). (M–P) Mks1 is normally found in the transition zone of the cilium (M, N), but it is absent from the cilium of *Wdpcp^Cys40^* mutant MEF (O, P). (Q–T) Wdpcp is localized to the transition zone in both control (Q, R) and *Mks1* mutant MEFs (S, T). All panels are at the same scales, and the scale bar in (A) is 2 µm.

Analysis of *Wdpcp^Cys40^* mutant embryos revealed ciliogenesis defects. This was observed in the kidney-collecting duct (Figure 2N,O,R) and in the neuroepithelium (Figure 2P,Q). Analysis of mouse embryonic fibroblasts (MEFs) derived from *Wdpcp^Cys40^* mutant embryos, referred to as *Wdpcp^Cys40^* mutant MEFs, confirmed a defect in ciliogenesis (Figure 2R; Figure S1A,B). Using an antibody raised to Wdpcp, we showed Wdpcp is localized to the ciliary axoneme and in a ring-like structure at the base of the cilia in IMCD3 cells (Figure 2D). A similar distribution was observed in NIH-3T3 cells transfected with Wdpcp–FLAG, a FLAG-tagged expression vector (Figure 2E–H). The Wdpcp ring-like structure in the cilium showed colocalization with Sept2, which is known to be present as a ring in the ciliary transition zone (Figure 2I–M; see Movie S2) [26]. In *Wdpcp^Cys40^* mutant MEFs, no specific Wdpcp immunostaining was observed, consistent with *Wdpcp^Cys40^* being a loss-of-function allele (see below). Even in rare *Wdpcp^Cys40^* mutant MEFs that are ciliated, Sept2 was usually not detected in the cilia (Figure 3I–L; 11 of 12 with no Sept2 staining; one with very low Sept2 staining). In contrast, wild-type MEFs typically showed strong Sept2 localization in the cilia (24 of 30 cilia).

To further interrogate the role of Wdpcp in ciliogenesis, we examined *Wdpcp^Cys40^* mutant MEFs for the distribution of Nphp1, a protein also found in the ciliary transition zone [29], and Mks1, a transition zone protein expressed only in the mother centriole and required for basal body docking to the membrane [27]. Both proteins are known to be required for ciliogenesis. Analysis of the rare ciliated *Wdpcp^Cys40^* mutant MEFs revealed Nphp1 was mislocalized to adjacent regions, such as in the basal body (Figure 3E–H; five of eight cilia), while Mks1 was absent (Figure 3M–P; 9 of 12 cilia). In comparison, Nphp1 (25 out of 27) and Mks1 (17 out of 21) were found in the cilia transition zone and basal body, respectively, of wild-type MEFs (*p*<0.01). These results show Wdpcp is required for recruitment of Mks1 and Nphp1 to the ciliary transition zone. However, Mks1 is not required for Wdpcp recruitment to the cilia, as *Mks1* mutant MEFs showed normal distribution of Wdpcp in the ciliary transition zone (Figure 3Q–T). In comparison, Ift88 distribution in the ciliary axoneme and basal body was unchanged in *Wdpcp^Cys40^* mutant MEFs, indicating IFT transport is not disrupted by Wdpcp deficiency (Figure 3A–D). Together, these observations suggest the ciliogenesis defect in *Wdpcp^Cys40^* mutant MEFs arises from the combined loss of Sept2, Nphp1, and Mks1 from the ciliary transition zone.

### Wdpcp Required for Motile Cilia Function in Zebrafish But Not Mouse Embryos

To examine whether Wdpcp also may play a role in motile cilia function, we examined cilia in the mouse embryonic node and in the trachea airway epithelium. The mouse embryonic node (Figure S1C,D) exhibited a normal pattern of ciliation with motile cilia (Movie S1) that generated effective flow (unpublished data). This is consistent with the absence of laterality defects in the *Wdpcp^Cys40^* mutants. Similarly, the trachea airway epithelia from E17.5 *Wdpcp^Cys40^* mutant embryos were ciliated, and the cilia were motile and exhibited normal cilia motility (Movie S3).

**Figure 4:**
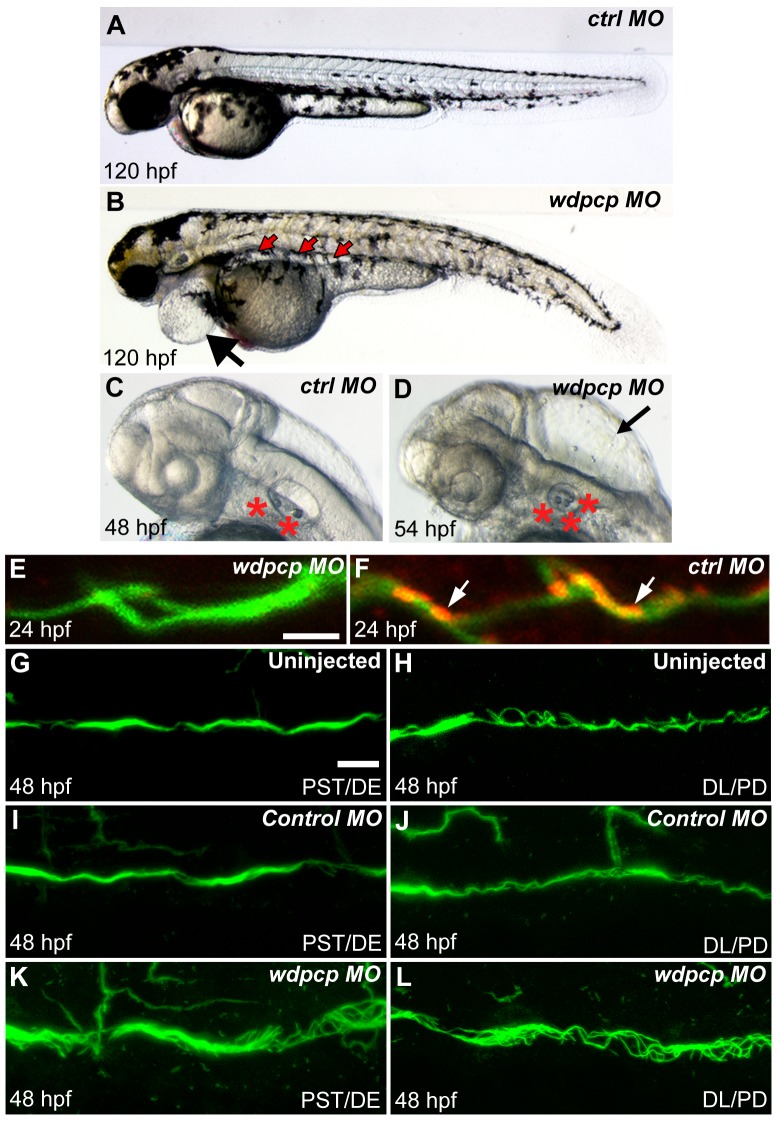
Ciliary defects in *wdpcp* morpholino knockdown embryos. (A–D) *wdpcp* morpholino knockdown causes pericardial edema (black arrow in B), pronephric tubule cyst (red arrows in B), severe hydrocephaly (arrow in D), and increased number of otoliths (red asterisks in D) as compared to control MO injected embryo (A). (E–L) Wdpcp antibody staining of the zebrafish embryo pronephric tubule showed punctate localization (red; arrows in F) along the ciliary axoneme (stained green with acetylated α-tubulin). Such staining is absent in *wdpcp* morphants, showing efficacy of the *wdpcp* MO knockdown (E). Cilia in proximal straight/distal early tubule (PST/DL) of *wdpcp* morphants are disorganized (K), as compared to that of control morphants (I) and uninjected embryos (G), while the distal late pronephric duct (DL/PD) showed little or no change compared to uninjected (H) or control MO (J) injected embryos. Scale bars, 2 µm in (E) and 10 µm (G). (E,F) and (G–L) are at the same scale.

In zebrafish, *wdpcp* is expressed starting from 10 somite stage (Figure S2A–O), and surprisingly, *wdpcp* morpholino (MO) knockdown resulted in a constellation of defects indicative of motile cilia defects. This included curved body axis (Figure 4B), pericardial effusion and kidney cysts (Figure 4B), hydrocephalus (Figure 4D), and increased number of otoliths (Figure 4D). Consistent with motile cilia defects, *wdpcp* morphants exhibited a 20% incidence of heterotaxy (Figure S3). *Wdpcp* morphants also exhibited kidney cysts (20%) and cilia disarray in the pronephric tubule, which were more pronounced proximally (Figure 4G–L). Videomicroscopy showed abnormal cilia motility throughout the pronephric tubule (Movie S4). As observed in the *Wdpcp* mouse mutants, some *wdpcp* zebrafish morphants (37%; *N* = 208) exhibited obstructed cloaca (Figure S3K–P). This was not correlated with the pronephric cilia disarray, as cilia disarray was observed in 38% of morphants with cloaca obstruction (*n* = 63) and 50% without cloaca obstruction (*n* = 82). Wdpcp antibody staining showed punctate Wdpcp localization in the ciliary axoneme in the pronephric tubule (Figure 4F), which was lost with *wdpcp* MO knockdown (Figure 4E). Western blotting confirmed Wdpcp protein expression is reduced with *wdpcp* MO knockdown (Figure S2P,Q). Specificity of the MO knockdown effects was confirmed with injection of *wdpcp* mRNA, which showed complete rescue of the defect phentoypes seen with *wdpcp* MO knockdown (Figure S4E,F).

These findings suggest that unlike mouse embryos, Wdpcp is required for motile cilia function in zebrafish embryos. This raises the question of whether the *Wdpcp^Cys40^* allele is a null mutation or if it might have residual function that could account for the differences between the mouse and fish phenotypes. To examine this question, we generated a *Wdpcp* knockout mouse model by gene targeting (Figure S5A). Breeding of mice carrying the knockout allele showed homozygous *Wdpcp* knockout mice died at birth with the same spectrum of developmental anomalies seen in *Wdpcp^Cys40^* mutants, including anopthalmia, cleft palate, heart outflow tract (OFT) septation defects, duplex kidney, limb polydactyly (Figure S5B–E), and multiple organ cysts. As in *Wdpcp^Cys40^* mutants, no laterality defects were noted. These findings demonstrate that the phenotypes observed in *Wdpcp^Cys40^* mutants reflect the loss of Wdpcp function.

### Disruption of Shh Signaling in *Wdpcp^Cys40^* Mutants

**Figure 5:**
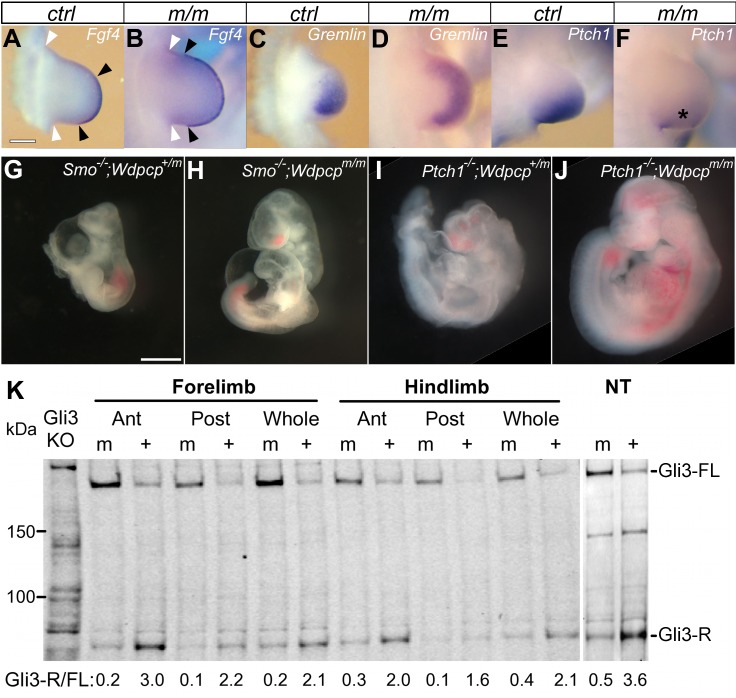
Sonic hedgehog signaling defect in *Wdpcp^Cys40^* mutant. (A–F) In-situ hybridization of E10.5 forelimbs shows *Wdpcp^Cys40^* mutants with expanded expression of *Fgf4* in the AER (apical ectodermal ridge) (A, B) and *Gremlin* in the limb mesenchyme (C, D), but reduced expression of *Ptch1* (E, F and asterisk in F). In (A) and (B), black arrowheads indicate the span of the AER, and white arrowheads are the anterior and posterior bases of the limb bud. (G–J) *Wdpcp* deficiency rescued the severe defect phenotypes of *Smo* ^−/−^ (G) and *Ptch1* ^−/−^ (I) mutant embryos at E10.5 dpc. The *Wdpcp^Cys40/Cys40^*;*Smo* ^−/−^ (H) and *Wdpcp^Cys40/Cys40^;Ptch1* ^−/−^ (J) double homozygous mutant embryos collected at E10.5 dpc show more robust growth with better axial development and also more normal head and heart development. (K) Western blotting of Gli3 in tissue extracts obtained from the limb and neural tube shows a decrease of Gli3-R/Gli3-FL ratio in *Wdpcp^Cys40^* mutant embryos. Scale bars, 200 µm in (A), 1 mm in (G). Scales are the same in (A–F) and (G–J).

Many of the *Wdpcp^Cys40^* mutant phenotypes, such as the limb polydactyly, TEF, and cleft palate, are consistent with the disruption of Shh signaling, which is known to be cilia transduced. Consistent with this, analysis of *Wdpcp^Cys40^* mutant MEFs showed little response to stimulation with SAG, a Shh agonist (Figure S6B). In situ hybridization analysis of the developing limb buds in *Wdpcp^Cys40^* mutants showed expansion of *Fgf4* and *Gremlin* expression in the apical ectodermal ridge and digit forming mesenchyme, respectively, while *Ptch1* expression was diminished (Figure 5A–F). These results confirmed the disruption of Shh signaling in the limb bud, consistent with the polydactyly phenotype. We also observed disruption of Shh signaling in the neural tube, which was indicated by dorsalization of the neural tube (Figure S6A). Further examination of *Wdpcp^Cys40/Cys40^*;*Smo* ^−/−^ (*n* = 3) and *Wdpcp^Cys40/Cys40^;Ptch1* ^−/−^ (*n* = 4) double mutant embryos showed rescue of the severe *Smo* and *Ptch1* knockout phenotypes (Figure 5G–J). This indicated *Wdpcp* functions downstream of *Smo* and *Ptch1*. Abnormal Gli transcription factor processing was revealed with Western blotting analysis of isolated limb bud and whole embryo extracts. This is indicated by alterations in the ratio of full-length activator versus shorter cleaved inhibitor Gli3 protein (Figure 5K; Figure S6C). We also observed more full-length Gli2 in the *Wdpcp^Cys40^* mutant embryos (Figure S6D).

### PCP Defects in *Wdpcp^Cys40^* Mutants

**Figure 6:**
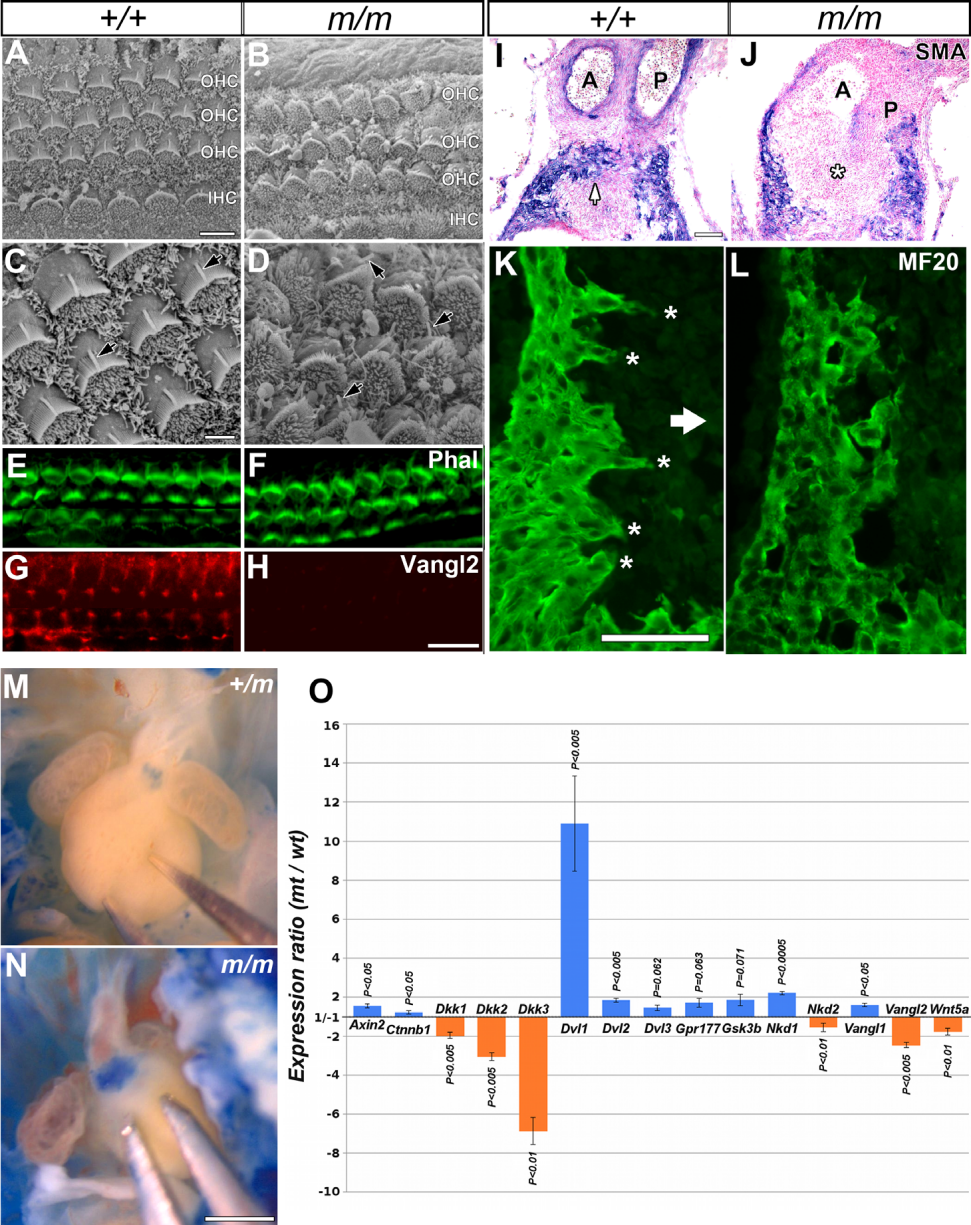
*Wdpcp^Cys40^* mutants show PCP defects and disrupted canonical Wnt signaling. (A–D) Scanning EM of cochlear hair cells showed chevron-shaped stereocilia pointing laterally in control (A, C), but in *Wdpcp^Cys40^* mutants, the stereocilia point in varying directions (B, D), with misshappened stereocilia bundles. The kinocilia, normally positioned at the chevron tip, were mislocalized in *Wdpcp^Cys40^* mutant. (E–H) Staining with phalloidin (Phal; E, F) and Vangl2 antibody (G, H) showed diminished Vangl2 expression in hair cells of *Wdpcp^Cys40^* mutant cochlea, while Vangl2 in control exhibited the characteristic asymmetric expression pattern. (I, J) Transverse sections of E13.5 embryos showed base of the heart counterstained with nuclear fast red and immunostained with anti α-SMA antibody (blue) to visualize migrating cardiomyocytes. In wild-type heart (I), cardiomyocytes were observed in the outflow tract cushion (arrow), but in *Wdpcp^Cys40^* mutants, cardiomyocytes were mostly absent in the cushion tissue (asterisk in J). (K, L) Cardiomyocytes in outflow cushion of wild-type embryos (K) visualized with MF20 immunostaining showed polarized cell morphology with distinct elongated finger-like projections (asterisks) aligned with direction of cell migration and projecting into forming outflow septum (arrow in K). In contrast, in *Wdpcp^Cys40^* mutant embryos (L), the cardiomyocytes exhibited rounded morphology without obvious cell polarity, nor the elongated cell projections seen in wild-type embryos. (M, N) The heart of control embryo at E13.5 showed very weak BAT–lacZ expression at the base of the pulmonary trunk (M), while markedly elevated BAT–lacZ expression was observed in the same region in a *Wdpcp^Cys40^* mutant embryo (N). (O) Quantitative real-time PCR analysis showed changes in expression level for noncanonical and canonical Wnt signaling pathway genes at the base of the outflow tract of *Wdpcp^Cys40^* mutant embryos. Blue bars indicate elevated versus orange bars, indicating reduced expression levels. Standard errors and *p* values are shown. Scale bars, 5 µm in (A, B), 2 µm in (C, D), 50 µm in (E–H), 100 µm in (I–L), 1 mm in (M, N).

We investigated *Wdpcp^Cys40^* mutants for PCP defects, given *Wdpcp* plays an important role in PCP regulated convergent extension cell movement in the *Xenopus* embryo [26]. We examined patterning of hair cells in the cochlea, as it is well described to be PCP dependent. The hair cells are normally arrayed in repeating rows, all exhibiting the same polarized orientation as defined by the actin-based stereocilia bundles. These are organized in a stereotypical “chevron” configuration, each with a single microtubule-based kinocilium protruding at the tip of the chevron (for review see [30]) (Figure 6A,C,E). Examination of the cochlea of *Wdpcp^Cys40^* mutants showed the hair cells were disarrayed, with some of the chevron misaligned (Figure 6B,F). Although the kinocilia were present, they were mislocalized (Figure 6D). We also observed expression of Vangl2, a membrane-localized PCP core component normally asymmetric expressed in the hair cells, was nearly extinguished in the *Wdpcp^Cys40^* mutant cochlea (Figure 6G,H).

*Wdpcp^Cys40^* mutants also exhibit outflow tract septation defects, another PCP-dependent developmental process. PCP is thought to regulate outflow tract septation via its role in modulating myocardialization of the outflow tract—a process in which cardiomyocytes invade and migrate into the conotruncal region of the heart to form the muscular outlet septum [31],[32]. In E13.5 control hearts, cardiomyocytes can be seen invading into the outflow tract at the base of the aorta and pulmonary trunk (Figure 6I), with the invading cells exhibiting an elongate morphology with long cell processes projecting into the outflow cushion aligned with the direction of cell migration (Figure 6K). However, in the *Wdpcp^Cys40^* mutant heart, cardiomyocytes failed to invade the OFT cushion (Figure 6J), and they did not exhibit the polarized cell projections seen in the wild-type heart (Figure 6L). These observations suggest polarized cell migration required for formation of the outflow septum in the embryonic heart is compromised in the *Wdpcp^Cys40^* mutant.

### Canonical Versus Noncanonical Wnt Signaling in *Wdpcp^Cys40^* Mutants

Using real-time PCR analysis, we further examined the expression of transcripts for components of the noncanonical Wnt signaling pathway known to regulate PCP using RNA obtained from the base of the OFT where the outlet septum forms (Table S1). This analysis showed a reduction in the expression of *Wnt5a*, a noncanonical Wnt ligand (Figure 6O). We also examined expression of the canonical Wnt signaling components, as opposing changes in noncanonical versus canonical Wnt signaling have been observed in ciliopathy mutants [24]. Indeed, real-time PCR analysis showed an increase in *Axin2* and *Dishevelled* (*Dvl1/2/3*) transcripts, while transcripts for the canonical Wnt inhibitors *Dkk1/2/3* were reduced (Figure 6O). Consistent with these real-time PCR results, analysis of *Wdpcp^Cys40^* mutants carrying the *BAT*–*lacZ* canonical Wnt reporter [33] also showed marked increase in lacZ expression at the base of the outflow tract (Figure 6M,N). Together, these observations suggest that while noncanonical Wnt signaling is reduced, canonical Wnt signaling is upregulated in the *Wdpcp^Cys40^* mutants.

### Wdpcp Modulation of Actin Stress Fibers

**Figure 7:**
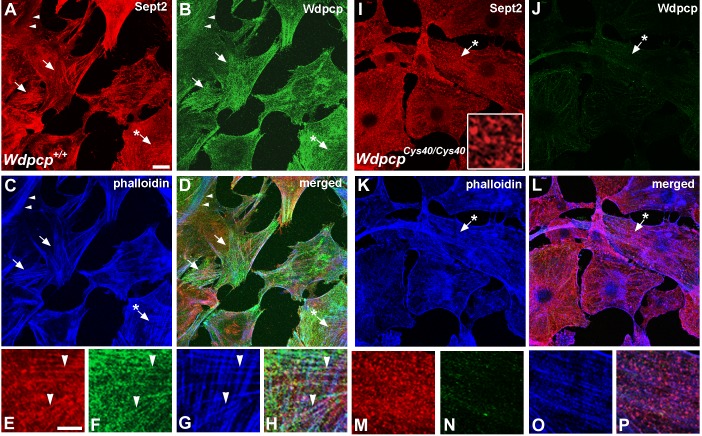
Wdpcp colocalizes with Sept2 and actin filaments. (A–D) Confocal imaging of control MEFs stained with phalloidin, and antibodies to Wdpcp and Sept2 showed Sept2 (red) and Wdpcp (green) are colocalized in actin stress fiber (phalloidin stained, blue) (examples denoted by arrow). This is better visualized in the magnified image shown in (E–H). (E–H) Magnified view of the region indicated by the asterisk-denoted arrow from (A–D) show colocalization of Wdpcp (green) and Sept2 (red) with actin filaments (blue). (I–L) Confocal imaging of *Wdpcp^Cys40^* mutant MEFs showed only background fluorescence (green, panel J) with the Wdpcp antibody. However, Sept2 immunostaining remained robust (red, panel I), but there was no colocalizaiton with actin filament visualized with phalloidin staining (blue, panel K). The region indicated by the asterisk-denoted arrow is magnified in (M–P). Inset shown is magnified view of a region from (M), showing ‘c’ and ‘o’ shaped Sept2 immunostained structures. (M–P) Magnified view of the region marked by the asterisk-denoted arrow in (I–L). While actin (blue, panel O) and Sept2 filaments (red, panel M) can be observed, Sept2 is not colocalized with actin (P). Sept2 immunostaining delineated ‘c’ and ‘o’ shaped structures (M), which are better visualized in the further magnified view shown in the inset in (I). Scale bars, 20 µm in (A), 10 µm in (E), and 5 µm in inset image in (I). Scales are the same in (A–D), (I–L); (E–H), (M–P).

To investigate the mechanism by which *Wdpcp* may regulate PCP, we further examined Wdpcp distribution in the cytoplasm, in particular its interaction with Sept2, which is known to associate with the actin cytoskeleton [34],[35]. Interestingly, in wild-type MEFs, Wdpcp showed extensive colocalization with actin filament bundles or stress fibers delineated by phalloidin staining (Figure 7A–H). Given the known role of Sept2 in associating with and stabilizing actin filaments, we investigated whether Wdpcp and Sept2 may be colocalized in actin filaments. Phalloidin staining to visualize actin filaments together with double immunostaining with Sept2 and Wdpcp antibodies indeed showed regions of Wdpcp and Sept2 colocalization in actin stress fibers in wild-type MEFs (Figure 7A–H). Similar analysis of the *Wdpcp^Cys40^* mutant MEFs revealed the loss of Wdpcp immunostaining, and interestingly, the actin cytoskeleton was markedly changed. None of the aligned stress fibers comprising of thick actin filament bundles were observed, but instead only thin actin filaments were seen (Figure 7J,N). Sept2 exhibited a beaded arrangement that were loosely aligned with but not colocalized with the actin filaments (Figure 7M,N,P). A magnified view showed these beaded structures were comprised of “o” and “c” shaped rings and circles similar to those previously reported in cells treated with latrunculin to disrupt the actin cytoskeleton [36]. Quantitation showed phalloidin staining was reduced by 28% in the mutant (*n* = 144) versus control (*n* = 111) MEFs (*p* = 0.011), consistent with the observed reduction in actin stress fibers. These observations suggest Wdpcp, through interactions with Sept2, may play an essential role in modulating actin filaments and the formation of stress fibers.

### Wdpcp and Sept2 Interaction

**Figure 8:**
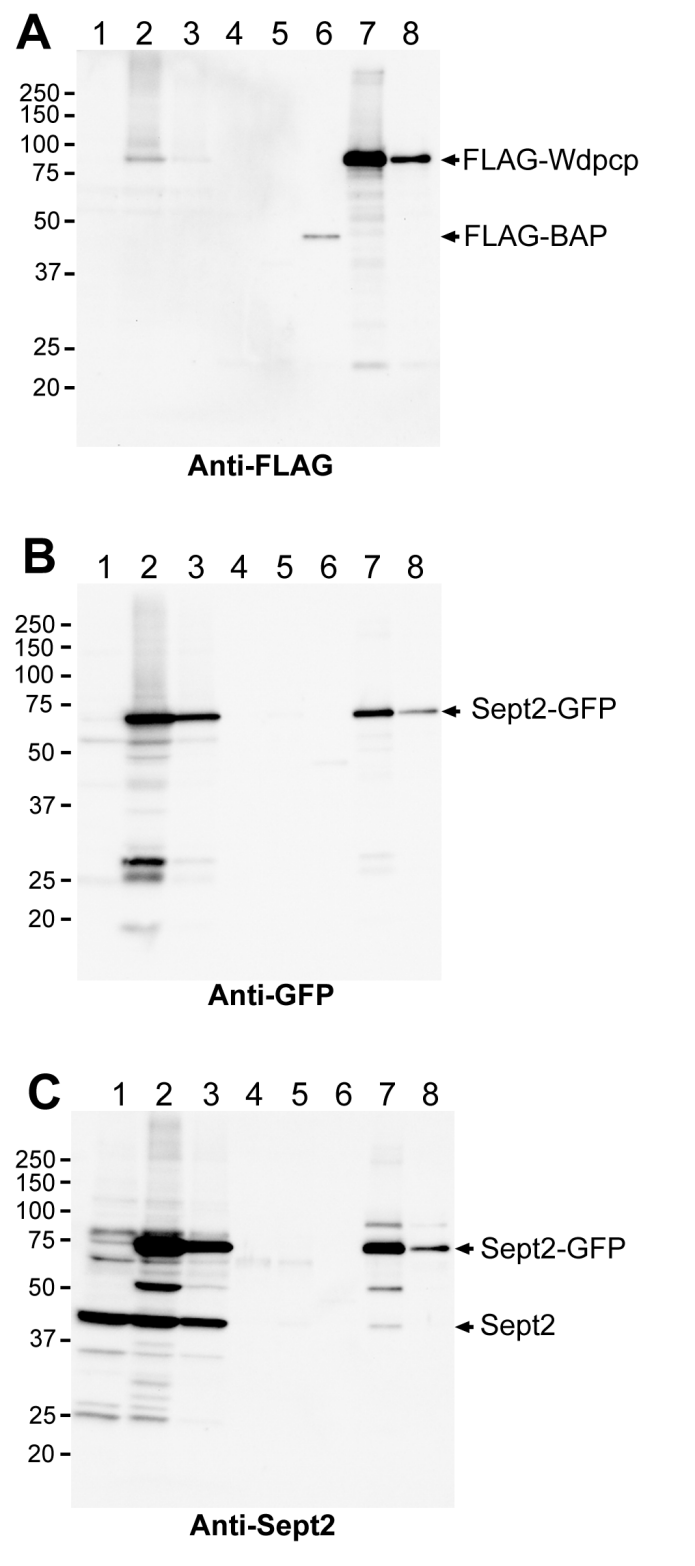
Analysis of Wdpcp and Sept2 interaction with immunoprecipitation and Western blotting. HEK-293 cells expressing FLAG-tagged Wdpcp and Sept2–GFP were lysed and immunoprecipitated with a FLAG antibody, and the immunoprecipitates were then analyzed by gel electrophoresis and Western blotted in triplicates for immunodetection with a FLAG (A), GFP (B), and Sept2 (C) antibody. FLAG-tagged Wdpcp was observed to coimmunoprecipitate with Sept2–GFP and Sept2 (lanes 7 and 8). The eight lanes included: untransfected whole cell lysate (lane 1), whole cell lysate of cells stably transfected with FLAG–Wdpcp and transiently transfected with Sept2–GFP (lane 2), whole cell lysate of cells transiently transfected with Flag–Wdpcp and Sept2–GFP (lane 3), blank immunoprecipitation elution control (lane 4), immunoprecipitation negative control of untransfected cell lysate (lane 5), FLAG immunoprecipitation of FLAG-bacterial alkaline phosphatase (lane 6), FLAG immunoprecipitation of cells stably transfected with FLAG–Wdpcp and cotransfected with Sept2–GFP (lane 7), and FLAG immunoprecipitation of cells transiently transfected with Sept2–GFP and FLAG–Wdpcp (lane 8).

To interrogate Wdpcp interaction with Sept2, we carried out coimmunoprecipitation experiments to determine if Wdpcp and Sept2 may be found in the same protein complex. For these experiments, HEK-293 cells that were either transiently or stably expressing FLAG-tagged Wdpcp (FLAG–Wdpcp) were transfected with Sept2–GFP. Western blotting of total cell lysates using an anti-FLAG antibody gave the expected 90 kDa (722 AA for Wdpcp plus 3× FLAG tag) in the stably transfected cells (lane 2, Figure 8A), but not in nontransfected cells (lane 1, Figure 8A). In the transiently transfected cells, a very weak band was detected only with long exposure, indicating very low protein abundance (lane 3 in Figure 8A; also unpublished data). This was confirmed with FLAG immunoprecipitation, which showed FLAG–Wdpcp was present in much lower abundance in the transiently transfected versus stably transfected cells (lane 7 versus 8 in Figure 8A).

When the same cell lysates were immunoblotted with anti-GFP antibodies, a 66 kDa Sept2–GFP fusion protein band (361 AA for Sept2 and 238 AA for GFP) was observed (lanes 2 and 3 in Figure 8B). Significantly, this band was also detected in the anti-FLAG immunoprecipitates (lanes 7 and 8, Figure 8B), indicating that Sept2–GFP and FLAG–Wdpcp are in the same protein complex. When the same cell lysates were immunoblotted with anti-Sept2 antibodies, the endogenous Sept2 band (∼40 kDa) was observed in all whole cell lysates (Sept2 band in lanes 1, 2, and 3 in Figure 8C), and in the anti-FLAG immunoprecipitates from cells stably transfected with FLAG–Wdpcp (Sept2 band in lane 7 in Figure 8C). These results indicate Sept2–GFP and endogenous Sept2 are both incorporated into a FLAG–Wdpcp-containing protein complex. The specificity of the FLAG immunoprecipitations were confirmed with positive control using FLAG-tagged bacterial alkaline phosphatase (FLAG–BAP band in lane 6 of Figure 8A), and negative control with nontransfected cells (lane 5 in Figure 8) or blank immunoprecipitation elution control (lane 4 in Figure 8). Together these results confirm that Wdpcp and Sept2 are recruited in the same protein complex.

### Wdpcp Modulation of Cell Motility and Focal Adhesion Contacts

**Figure 9:**
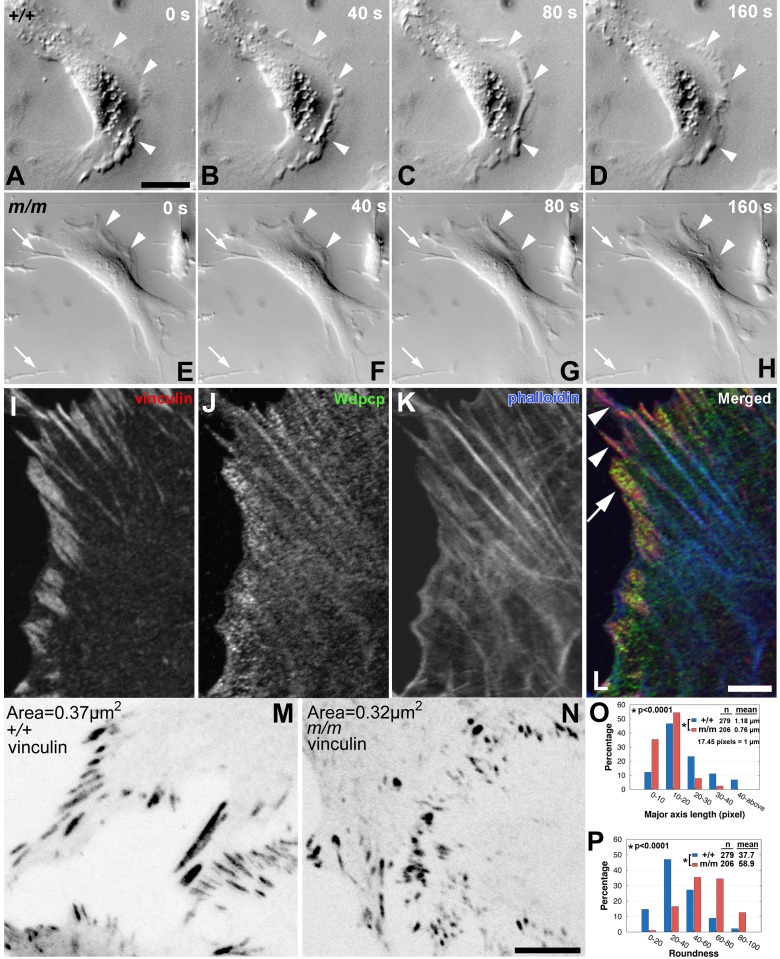
Perturbation of membrane ruffling and focal adhesion contacts in *Wdpcp*-deficient cells. (A–H) Time-lapse imaging of wild-type (A–D) and *Wdpcp^Cys40^* mutant (E–H) MEFs shown in 40 s intervals revealed the mutant MEFs have less membrane ruffling (arrowheads) and little or no membrane protrusive activity. In (E–H), the bottom-left arrow points to a long filopodial extension from a cell out of the field of view and top-left arrow points to the filopodial extension of the cell in the field of view that were immobile for the entire duration of the 320 s time-lapse sequence. (I–L) Immunostaining showed Wdpcp (green, J) is enriched at the cell cortex where actin filaments (phalloidin, K) insert into vinculin- (red, I) containing focal adhesions (arrow and arrowheads in L). (M, N) In *Wdpcp^Cys40^* mutant MEFs (N), the vinculin-containing focal adhesions were smaller and more rounded as compared to wild-type MEFs (M). This was demonstrated by quantitative measurements of the major axis and roundness of vinculin-containing focal adhesions (O, P). (O, P) In wild-type MEFs (*n* = 279), the vinculin-containing focal contacts were more elongated (O) and less round (P), than *Wdpcp^Cys40^* mutant MEFs (*n* = 206). The *p* values were calculated with student's *t* test. Scale bars, 10 µm in (A), 2 µm in (L), and 10 µm in (N). Scales are the same in (A–H); (I–L); and (M, N).

To examine the role of Wdpcp in actin dynamics and motile cell behavior, we carried out time-lapse videomicroscopy to assess cell motility in the *Wdpcp^Cys40^* mutant and wild-type MEFs. Time-lapse imaging showed dynamic membrane ruffling with lamellipodial and/or filopodial cytoplasmic extensions in wild-type MEFs (Figure 9A–D). In contrast, *Wdpcp^Cys40^* mutant MEFs exhibited minimal membrane ruffling activity (Figure 9E–H, arrowheads) and often with unusually long and thin filopodial extensions that appeared unable to disengage from the substratum (Movie S5) (see arrows in Figure 9E–H). This was confirmed with quantitative analysis of the time-lapse videos, which showed the mutant MEFs were more likely to have filopodia (46.03% versus 19.99% in wild-type MEFs; *p*<0.0001), and the filopodia persisted longer (3.0 h versus 1.12 h in wild-type MEFs; *p*<0.0001). To assess membrane ruffling, we examined the frequency of brightness change at the cells' leading edge and calculated the fluctuation period as a measure of membrane dynamics (see Materials and Methods). This analysis showed significantly less membrane ruffling in the mutant MEF, which had a fluctuation period of 272 s versus 167 s for wild-type MEFs (*p*<0.0002).

As cell motility requires actin remodeling coordinated with the assembly/disassembly of focal contact, we further examined the role of Wdpcp in the modulation of focal adhesion contacts. Phalloidin, anti-Wdpcp, and anti-vinculin triple staining showed Wdpcp is enriched in the cell cortex, where it is extensively colocalized with vinculin at points of actin filament insertion into focal adhesions (Figure 9I–L). While wild-type MEFs exhibited elongated focal contacts, focal contacts in mutant MEFs appeared to be smaller and more round in shape (Figure 9M,N). Quantitative analysis showed the average size of focal adhesion contacts was decreased in the mutant MEFs (0.37 µm^2^ versus 0.32 µm^2^, *p*<0.001; Figure 9M,N). However, the mean intensity of vinculin immunostaining was significantly increased (157.8 in the *Wdpcp^Cys40^* mutant versus 100.2 in wild-type MEFs; *p*<0.01; grayscale intensity range, 0–255). The focal adhesion contacts exhibited a reduction in length (0.76 µm in *Wdpcp^Cys40^* mutant versus 1.18 µm in wild-type MEFs; *p*<0.0001; Figure 9O), but increase in roundness (58.9 in *Wdpcp^Cys40^* mutant versus 37.7 in the wild-type MEFs, *p*<0.0001; Figure 9P). These marked changes in the organization of the focal adhesion contacts may contribute to the defects in cell motility and directional cell migration observed in *Wdpcp^Cys40^* mutant MEFs.

### Wdpcp Modulates Polarized Cell Migration

**Figure 10:**
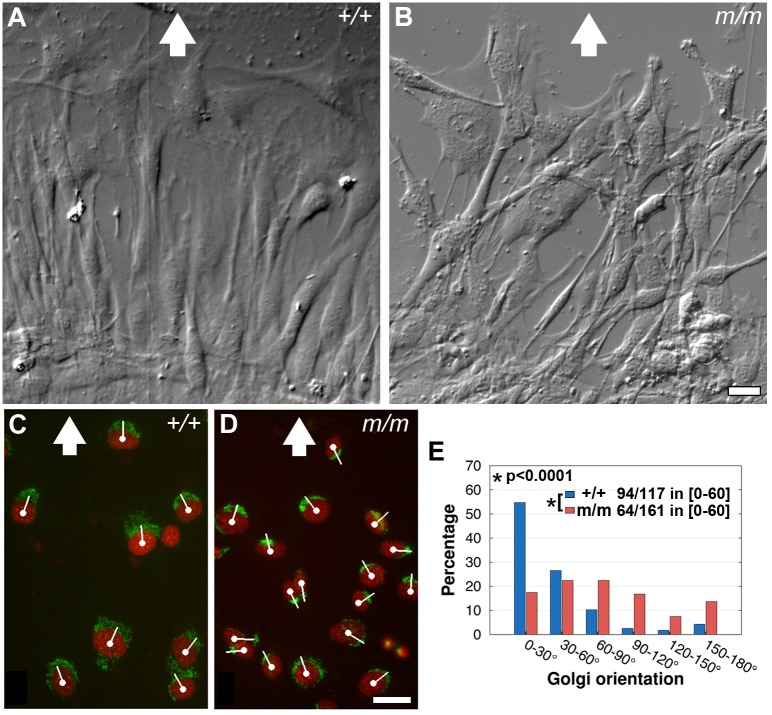
*Wdpcp* deficiency disrupts polarized cell migration. (A–D) In a wound healing assay, control MEFs (A) were well aligned with the direction of wound closure (indicated by white arrow). In contrast, *Wdpcp^Cys40^* mutant MEFs (B) showed a disorganized distribution and with many long thin filopodial extensions. These differences in cell polarity were also reflected in their Golgi orientation—Golgi orientation is indicated by a white line drawn from the cell nucleus through the center of the Golgi (C, D). In wild-type MEFs, the Golgi was mostly situated at the cell's leading edge (C), aligned with the direction of wound closure (white arrow). However, in *Wdpcp^Cys40^* mutant MEFs, Golgi orientation was randomized (D). (E) Quantitative analysis of Golgi orientation showed wild-type MEFs (*n*=117) with highly polarized arrangement of the Golgi well aligned with the direction of cell migration (94 of 117 cells with Golgi apparatus in 0–60° sector), while *Wdpcp^Cys40^* mutant MEFs (*n*=161) showed random orientation of the Golgi apparatus (64 of 161 cells with Golgi in 0–60° sector) (see Materials and Methods). Scale bar, 20 µm in (B) and (D). Scales are the same in (A, B) and (C, D).

To investigate the role of Wdpcp in polarized cell migration, we carried out a wound-scratch assay to examine the ability of the *Wdpcp^Cys40^* mutant MEFs to engage in directional cell migration to fill the wound gap. For this analysis, wild-type and *Wdpcp^Cys40^* mutant MEFs were grown to confluence, and then a wound gap was created to assess directional cell migration required for wound closure. Wild-type MEFs migrated into the wound gap in a highly organized manner with cells aligned with the direction of wound closure (Figure 10A). In contrast, mutant MEFs migrated in a haphazard manner with no consistent cell alignment (Figure 10B). Immunostaining with a Golgi marker showed the Golgi apparatus in wild-type MEFs were localized at the cells' leading edge and were forward facing relative to the direction of wound closure (0–60° in Figure 10C,E), consistent with polarized cell alignment and directional cell migration. In contrast, in the mutant MEFs, the direction of Golgi orientation was randomized (Figure 10D,E). These observations show Wdpcp is required for establishing the planar cell polarity needed to engage in directional cell migration.

## Discussion

We showed *Wdpcp^Cys40^*, a mouse mutant with a wide spectrum of developmental defects consistent with MKS/BBS ciliopathy syndromes, harbors a *Wdpcp* loss of function mutation. We generated a *Wdpcp* knockout mouse model, which exhibited identical phenotypes to those seen in the *Wdpcp^Cys40^* mutants. A role for *Wdpcp* in human disease is suggested by a previous finding of a homozygous *WDPCP* mutation in a screen of MKS/BBS patients [26]. We observed Wdpcp deficiency disrupted ciliogenesis and this was associated with the disruption of Shh signaling, accounting for many of the defect phenotypes observed in the *Wdpcp^Cys40^* and *Wdpcp* knockout mice.

The *Wdpcp^Cys40^* mutant mice exhibited phenotypes that are remarkably similar to those observed in a MKS mutant mouse model, *Mks1^del64-323^* [27]. While heterotaxy was observed in the *Mks1* mutant, no laterality defects were found in the *Wdpcp^Cys40^* mutants. Consistent with this, nodal cilia motility and nodal flow were unaffected in *Wdpcp^Cys40^* mutant embryos. Surprisingly, *wdpcp* MO knockdown in zebrafish embryos did not disrupt ciliogenesis, but perturbed motile cilia function. This was associated with a low incidence of heterotaxy. It is interesting to note that *wdpcp* MO knockdown in *Xenopus* embryos also perturbed motile cilia function [26]. These species differences may reflect evolutionary divergence in the function of Wdpcp and other cilia-related proteins.

### Wdpcp Regulates Ciliogenesis via Recruitment of Ciliary Proteins

Our findings indicate the ciliogenesis defect in *Wdpcp^Cys40^* mutants arises from the failure of *Wdpcp*-deficient cells to recruit proteins required for ciliogenesis to the ciliary transition zone, including Nphp1, Sept2, and Mks1. Wdpcp is observed to form a ring structure in the ciliary transition zone overlapping with Sept2. Septins are well described to form ring structures both in vitro and in vivo [37]. In the ciliary transition zone, the septin ring forms a diffusion barrier regulating protein trafficking into the ciliary compartment [38]. Thus, failure to recruit Sept2 to the transition zone in *Wdpcp*-deficient cells is expected to disrupt ciliogenesis. Wdpcp-deficient cells also failed to recruit Mks1, a Meckel syndrome–associated protein localized to the mother centriole and required for basal body docking to the membrane [27],[28]. Analysis of Mks1-deficient cells showed Wdpcp acts upstream of Mks1. We also observed Wdpcp-deficient cells with mislocalization of Nphp1, a cilia transition zone protein required for ciliogenesis and associated with cystic kidney defects in Joubert syndrome and other ciliopathies [39]. Together these findings indicate Wdpcp may play an important role in recruiting proteins essential for ciliogenesis. Consistent with this is the presence of two WD repeats in the Wdpcp protein. The WD40 domain has been identified in many protein interaction pairs [40], and many WD40 repeat-containing proteins have been shown to serve as scaffolds for assembly of multiprotein complexes. Together these findings suggest Wdpcp may serve as a scaffold in the cilia transition zone to facilitate the assembly of multiprotein complexes required for ciliogenesis.

### *Wdpcp* Constrains Hedgehog and Canonical Wnt Signaling

While our studies with *Wdpcp^Cys40^* mutant MEFs showed Wdpcp deficiency disrupted Shh signaling, surprisingly the loss of Wdpcp function partially rescued the severe defect phenotypes of the *Ptch1* or *Smo* knockout embryos. This suggests *Wdpcp* may constrain Shh signaling downstream of *Smo*/*Ptch1*. As ciliogenesis is disrupted with Wdpcp deficiency, how Gli processing required for Shh activation is regulated in the *Wdpcp^Cys40^/Ptch1* or *Wdpcp^Cys40^/Smo* knockout embryos is unknown. However, it is worth noting *Drosophila* hedgehog signaling occurs in the absence of the cilium [41].

Our studies also suggested Wdpcp may have a role in constraining canonical Wnt signaling, as BAT–lacZ and the expression of canonical Wnt transducers (*Dvl1/2/3*) are upregulated, while canonical Wnt inhibitors were down-regulated (*Dkk1/2/3*) in the *Wdpcp^Cys40^* mutant OFT. While a role for the cilium in constraining canonical Wnt signaling is well described, the mechanism remains unclear [18]. We note Chibby, a basal body protein that negatively regulates canonical Wnt signaling, can bind β-catenin and prevent its entry into the nucleus [42]. One possibility to consider is whether Wdpcp may function in the same multiprotein complex with Chibby to regulate β-catenin trafficking.

### Wdpcp Modulates PCP and the Actin Cytoskeleton

We showed *Wdpcp^Cys40^* mutants exhibited PCP defects, such as malpatterning of stereocilia in the cochlea and abnormal myocardialization of the outflow tract in the heart. The myocardialization defect was characterized by failure of the invading cardiomyocytes to elongate and align their actin cytoskeleton with the direction of cell migration. We note the *Loop-tail* (*Lp*) mouse mutant harboring a mutation in the PCP component, *Vangl2*, also exhibits outflow tract defects [31]. Similar to our findings, in the *Lp* mutant cell protrusions into the outflow cushion were absent, and the organization of the actin cytoskeleton was disrupted. Perturbation of the actin cytoskeleton also may contribute to defects in the cochlea, as the stereocilia are actin-based structures and in *Wdpcp^Cys40^* mutants, formation of the kinocilia was not affected. While cochlea expression of Vangl2 was reduced in *Wdpcp^Cys40^* mutants, we did not observe mislocalization of PCP core components that would suggest a disruption in PCP signaling.

Using Wdpcp-deficient MEFs, we investigated the role of Wdpcp in establishing planar cell polarity. Our studies indicate Wdpcp plays an essential role in PCP via regulation of the actin cytoskeleton. We showed Wdpcp modulates the actin cytoskeleton by mediating Sept2 interaction with actin. In wild-type cells, Wdpcp is colocalized with Sept2 in actin filaments, but in Wdpcp-deficient cells, Sept2 is no longer actin localized and actin stress fibers are largely absent. Instead, Sept2 is observed in “o” and “c” configurations, structures also observed with inhibition of actin polymerization [36]. These results suggest formation of a Wdpcp–Sept2 complex may be required for Sept2 binding to actin filaments and the stabilization of actin filaments. Coimmunoprecipitation and Western immunoblotting confirmed Wdpcp and Sept2 localization in the same protein complex, but further experiments are needed to examine Wdpcp and Sept2 interaction with actin filaments.

A further indication of a role for Wdpcp in the modulation of actin dynamics was the reduction in membrane ruffling in Wdpcp mutant MEFs. We noted Wdpcp is enriched in the cell cortex where actin filaments insert into vinculin-containing focal adhesions. As focal contacts are also sites of actin polymerization, the abundance of Wdpcp in the vicinity of focal contacts may facilitate recruitment of Sept2 to enhance actin filament stabilization and stress fiber formation. We observed Wdpcp-deficient cells had smaller focal contacts, but with significantly higher concentration of vinculin. This could account for the failure of cell processes to disengage from the substratum in Wdpcp-deficient cells.

Most significantly, Wdpcp-deficient cells showed defects in motile cell behavior that was associated with defects in planar cell polarization. Thus, Wdpcp-deficient cells were unable to establish cell polarity in a wound scratch assay. This is indicated by failure of the Golgi to reorient to the cell's leading edge and align with the direction of cell migration. As a result, migrating cells were unable to engage in directional cell motility. It is significant to note that Golgi orientation is specified by the microtubule-organizing center or centrosome, which also templates formation of the cilium. This defect in establishing cell polarity together with perturbation in membrane and actin dynamics and the modulation of focal adhesion contacts may underlie the PCP defects associated with Wdpcp deficiency. Together, these findings suggest Wdpcp may regulate planar cell polarity by modulating both the microfilament and microtubule cytoskeleton.

### Dual Functionality of Wdpcp in Ciliogenesis and PCP

We showed the PCP effector Wdpcp plays an essential role in both ciliogenesis and PCP. This appears to involve separable functions of Wdpcp in recruitment of proteins to the ciliary transition zone required for ciliogenesis, and in the modulation of actin dynamics in the cytoplasm. Our findings provide the first evidence, to our knowledge, that a PCP component required for ciliogenesis can directly modulate the actin cytoskeleton to regulate planar cell polarity and directional cell migration. These observations suggest Wdpcp regulation of PCP is independent of its role in ciliogenesis. It is interesting to consider whether such dual functionality may have evolved as a means to functionally integrate pathways regulating ciliogenesis with those regulating PCP and the cytoskeleton. Whether such dual functionality may account for other PCP proteins known to be required for ciliogenesis is an important question that needs to be addressed in future studies.

## Materials and Methods

### Institutional Approval for Animal Studies

All mouse experiments were carried out using protocols approved by the Institutional Animal Care and Use Committee at the National Heart Lung Blood Institute and at the University of Pittsburgh. The zebrafish experiments were carried out with approved protocols at the University of Pittsburgh.

### Mapping and Recovery of the *Wdpcp* Mutation and Mouse Breeding

C57BL/6J(B6)/C3H hybrid mutant offspring were used to map the mutation using 48 microsatellite markers polymorphic between B6/C3H [43]. Once the chromosome interval was identified, additional polymorphic DNA markers were used to narrow the interval to a 5–10 Mb region. Then cDNA sequencing was carried out for each gene in the interval.

*Smo^tm1Amc^* (strain 004288), *Ptch1^tm1Mps^* (strain 003081), and *BAT–lacZ* (strain 005317) mouse lines were obtained from the Jackson Laboratory and intercrossed with the *Wdpcp^Cys40^* mouse line to generate double heterozygous mice, which were further intercrossed to generate embryos double homozygous for the *Wdpcp^Cys40^* and *Smo^tm1Amc^*, *Wdpcp^Cys40^* and *Ptch1^tm1Mps^*, or homozygous *Wdpcp^Cys40^* carrying *BAT–lacZ* allele.

### Construction of FLAG-Tagged Wdpcp Construct

To make the mouse FLAG–Wdpcp construct, we isolated RNA from mutant heart and amplified the full-length cDNA using one step RT-PCR using Superscript III/HF Platinum Taq DNA polymerase and cloned cDNA into pCR2.1 TOPO vector. We then confirmed the clones by Sanger sequencing. We amplified TOPO vector containing Wdpcp (NM_145425.3) cDNA using primers (Table S1) with restriction site at the 5′ end, cut with appropriate restriction endonuclease, and cloned into expression vectors containing FLAG at either the 5′ end or 3′ end (Sigma-Aldrich). The subsequent experiments were performed by using the FLAG at the 5′ end of Wdpcp.

### Zebrafish *wdpcp* Morpholino Knockdown and Rescue

Zebrafish embryos were obtained from incrossing wild-type adults and following manipulation, maintained at 28.5°C, and staged according to [44]. A complementary MO (5′-AGCTCCGCCAGGCAGAACGACATCT-3′) targeting the initiation codon of zebrafish *wdpcp* and standard control (5′-CCTCTTACCTCAGTTACAATTTATA-3′) morpholino (MO) were designed and obtained from GeneTools LLC. Embryos at the one-cell stage were injected with 5 nl of morpholino at 2.0 ng/nl in phenol red. Control embryos were either injected with the standard control (2.0 ng/nl) MO or uninjected. Capped mRNAs for rescue experiments were synthesized *in vitro* from linearized pCS2^+^ mouse Wdpcp construct (NM_145425.3) using SP6 mMessage mMachine kit (Ambion). We microinjected 200 pg of *Wdpcp* mRNA at 200 pg/1 nl into the blastomere at the one-cell stage. After injections, the embryos were incubated in1×E3 medium (5 mM NaCl, 0.17 mM KCl, 0.33 mM CaCl_2_, 0.33 mM MgSO_4_, 0.01% methylene blue) at 28°C until the desired stage and imaged using Leica MZ16 microscope fitted with Retiga 1300 camera. Statistical distribution of morphants and morphologically normal embryos from three separate rescue experiments were analyzed using Graphpad prism version 6.

### Zebrafish in Situ Hybridization and Immunocytochemistry

For in-situ hybridization, zebrafish embryos were fixed in 4% paraformaldehyde and processed as described [45]. Partial zebrafish *wdpcp* cDNA clone obtained from OpenBiosystems (#EDR1052-97951134, clone ID 7911360) was used to generate sense and antisense in-situ probes for *wdpcp*. Plasmids for *cmlc2* and *fabp10a* in-situ probes were obtained from members of the zebrafish community and were described previously [46],[47]. For immunocytochemistry, zebrafish embryos were fixed in Dent's fixative (80% Methanol and 20% DMSO) for 4 h at room temperature, washed with methanol, and stored at −20°C. Antibody staining for acetylated α-tubulin was performed as described [48]. For Wdpcp and acetylated α-tubulin double immunostaining, embryos were rehydrated in a series of methanol/PBT washes and permeabilized with 0.02% trypsin for 5 min. Following blocking with 10% normal goat serum (NGS, Sigma), embryos were incubated overnight at 4°C in PBT/10% NGS with Wdpcp (1∶100; Aves, Inc) and acetylated α-tubulin (1∶1,000; Sigma) antibodies. Goat anti-mouse Alexa Fluor 488 and goat anti-chicken Alexa Fluor 555 (both 1∶1,000; Invitrogen) secondary antibodies were used. Embryos were de-yolked, mounted in Aqua Poly/Mount (Polysciences, Inc.), and visualized with a Zeiss LSM 510 Meta inverted laser scanning confocal microscope.

### Production of *Wdpcp* KO Mice

The *Wdpcp* target or null allele was generated by gene targeting in 129 ES cells, as shown in Figure S3A. Exon 5 was flanked by two loxP/Flox sites and an FRT-flanked PGKneo cassette (neomycin-resistant gene driven by the PGK promoter) was inserted in intron 5 of *Wdpcp* by homologous recombination. ES cell clones with correct homologous recombination were screened by long-range PCR. Two independent ES cell clones were injected into C57BL/6J blastocysts, and germ line transmission was achieved with both clones. The PGKNeo cassette was removed by FLP-FRT recombination to generate *Wdpcp* flox allele. The *Wdpcp* global knockout allele was generated by CMV-Cre/Lox recombination.

### Immunocytochemistry and Immunohistology

MEFs derived from E11.5–E12.5 embryos were serum starved for 24 to 48 h to grow out the cilia, with staining of ciliary proteins carried out using various antibodies including acetylated α-tubulin antibody (Sigma T7451, 1∶1,000), γ-tubulin antibody (Sigma T6557, 1∶1,000), Wdpcp chicken polyclonal antibody (Aves, Inc.) made against synthetic peptide (DTTILEYREPVSKYARR) corresponding to Wdpcp amino residues 529–545, Wdpcp goat antibody (Santa Cruz), Sept2 antibody (Millipore, 1∶1,000), Mks1 antibody (1∶2,000) [27], and antibodies to Ift88 and Nphp1 as previously described [49],[50]. FLAG antibody (Sigma F7425, 1∶1,000) was used for detection of Wdpcp–FLAG fusion protein.

Kidney paraffin sections were stained with DBA (*Dolichos biflorus* agglutinin, Sigma, 1∶20) to delineate kidney tubules. Dissected cochleae were stained with Phalloidin and Vangl2 antibody as described previously [12]. Heart cryo-sections were stained with antibodies to MF20 (Hybridoma Bank, 1∶40), smooth muscle actin (Sigma, 1∶40). Neural tube cryo-sectioning and immunostaining with FoxA2 (Hybridoma Bank, 1∶1,000), Pax6 (Hybridoma Bank, 1∶1,000), and Olig2 (gift from Dr. Bennett Novitch at UCLA, 1∶1,000) was previously described [27].

### Analysis of Shh Signaling

DIG-labeled probes were made with DIG RNA reaction mixture (Roche), and procedures for whole mount in-situ hybridization were described previously [51] and gene expression patterns were visualized with BM purple AP substrate (Roche). RNA in-situ probe plasmids for *Fgf4* (Lee Niswander lab), *Gremlin* (Richard Harland lab), and *Ptch1* were gifts from Dr. Susan Mackem (NCI). A 600 bp nucleotide *Gli3* in-situ probe was generated by amplifying cDNA specific to Gli3 encompassing exons 12–14.

For limb Gli3 Western blotting, left fore- and hindlimbs at E10.5 (32–37 somite stages) were harvested and bisected into anterior and posterior halves, whereas the right fore- and hindlimbs were harvested intact. The remaining tissues were further removed until the neural tube was left, which was then used for Gli2 and Gli3 Western blotting. In addition, whole mutant and wild-type E10.5 embryos were lysed and processed for Gli3 Western blotting. The Western blotting procedures for Gli2 and Gli3 were carried out as described previously in [27]. Gli2 and Gli3 antibodies were gifts from Dr. Baolin Wang at Cornell University and Dr. Susan Mackem at NCI, respectively.

### Quantitative Analysis of Vinculin-Containing Focal Adhesions

Focal adhesions were visualized using a vinculin antibody (Sigma V9264, 1∶1,000). The area of vinculin immunostaining was traced using the image editor Gimp (www.gimp.org), and the area and intensity of staining were measured in 8-bit images. To quantify the length and roundness of vinculin-containing focal adhesion sites, we measured the major axis (MA) and minor axes (MI) of each focal adhesion. The mean MA length was determined and was plotted as a histogram with bin size of 10 pixels (equivalent to 0.57 µm). The roundness factor for each focal adhesion contact was measured using MI/MA*100, with 100 being focal adhesions that were perfectly round with major and minor axes of equal length.

### Scanning EM and Videomicroscopy of Nodal Cilia and Pronephric Duct

Scanning EM of the nodal cilia and neural tube epithelium was carried out as described previously [27]. Analysis of nodal cilia motility in E8.0 mouse embryos and pronephric duct of zebrafish embryos were carried as described previously [52]. The videos were converted into Quicktime movies.

### Time-Lapse Videomicroscopy for Assessing Cell Motile Behavior

Time-lapse videomicroscopy was carried out to quantify the motile behavior of wild-type and *Wdpcp* mutant MEFs using an inverted microscope (Leica, DMIRE2) and a Hamamatsu ORCA-ER camera. Video sequences encompassing an 8 h recording period with images captured every 10 min were used to measured the number of cells having one or more filopodia/total number of cells (percentage of cells with filopodia), and we also measured the number of frames in which the same filopodia was observed (persistence of filopodia). To quantify membrane ruffling activity, we recorded cultured cells at a shorter time-lapse interval comprising 10 s for a total of 30 min. These time-lapse images were then used to measure the frequency of brightness change at the cells' leading edge to quantitate the membrane ruffling activity. This entailed selecting boxed regions from the leading edge of a migrating cell, summing the brightness for each frame, and performing Fourier transform to obtain the frequency of brightness changes. The inverse of this frequency provided an index of the “period” of membrane fluctuation (MFP, in seconds), which is a measure of membrane dynamics, with greater membrane ruffling indicated by lower MFP.

### Wound Scratch Assay for Assaying Polarized Cell Migration

Confluent MEF cultures were scratched using a 10 µl micropipette tip to generate a gap. Then time-lapse imaging was carried out using a 40× objective on an inverted microscope (Leica, DMIRE2) with images captured every 10 s over a 30 min interval using a Hamamatsu ORCA-ER camera. To examine cell polarity, cells were immunostained with a Golgi antibody (Sigma HPA021799, 1∶1,000) followed by DAPI staining. Cells that are polarized and aligned with the direction of migration have Golgi situated in front of the nucleus (forward facing) and aligned with the migration direction. Polarity was scored by overlaying a clock face on each cell, and polarized cells are defined as those with Golgi situated within a 60° sector centered along the direction of wound closure [53],[54].

## Supporting Information

Figure S1 **Ciliogenesis defect in** ***Wdpcp^Cys40^*** **mutant.** (A, B) Immunostaining of cilium with acetylated α-tubulin, a-tub (red), and γ-tubulin, (g-tub, green) antibodies showing a shorter cilium in *Wdpcp^Cys40^* mutant MEF (B). Scanning EM images of embryonic nodes of control (C) and mutant (D) embryos at E8.0 showing the node cells are ciliated normally and cilia in mutant embryonic node are of normal shape and length. Scale bars, 2 µm in (A) and (C). Scales are the same in (A, B) and (C, D).
(JPG)

Figure S2 ***Wdpcp*** **zebrafish in situ hybridization and morphant at 48 h, Western blotting with Wdpcp chicken antibody.** (A–O) Embryonic *wdpcp* mRNA localization (purple) by whole mount in-situ hybridization with *wdpcp* antisense riboprobe at the four-cell stage (A, B), eight-cell stage (C, D), 1,000-cell stage (E, F), and shield stage (G) showed absence of maternal *wdpcp* transcripts. Embryonic *wdpcp* expression is observed at the 10-somite stage (H, I) and at 24 hpf (J, K). At 48 hpf (L–O) *wdpcp* staining appears less specific, since faint staining is observed with both the sense and antisense probes. (P) Immunoblot using wdpcp antibody (green) with 24 hpf zebrafish embryo lysate showed effective knockdown of wdpcp protein expression. α-Tubulin (red) was used as a sample loading control. Lane 1, protein molecular weight markers; lane 2, lysate from embryos injected with 10 ng control morpholino (MO); and lane 3, lysate from embryos injected with 10 ng *wdpcp* morpholino. (Q) The ratio of wdpcp (green) to α-tubulin (red) in the immunoblot was quantified using Image studio version 2.0 from LI-COR Biosciences (Lincoln, NE), which showed significant reduction in the wdpcp protein with *wdpcp* MO knockdown. Scale bars, 200 µm in (A), (G), (I), (J), and (L) and 150 µm in (M). Scales are the same in (A–F), (H, I), (J, K), (L, N), and (M, O).
(JPG)

Figure S3 **Laterality defects in** ***Wdpcp*** **zebrafish morphants.** (A–D) Ventral view of RNA in-situ hybridization staining with *cmlc2* probe delineating the heart tube in 54 hpf embryos in *wdpcp* morphants revealed normal right-sided looping (B), no looping (C), or reversed heart looping (D) orientation. (E–H) Dorsal view of gut orientation as observed with *LFABP* in-situ hybridization analysis delineating liver position in 54 hpf embryo. Three types of gut orientation were observed: normal left-sided (F), duplicated (G), and right-sided (H). (I, J) Distribution of heart (I) and gut (J) looping orientation in *Wdpcp* morphants, with asterisk indicating statistically significant differences between control versus *wdpcp* morphants. (K–P) In-situ hybridization with an *evx1* probe on 24 hpf embryos (K–M) delineated the normal cloaca in uninjected (K) and control MO (L) injected embryos, while in the *wdpcp* morphant (M), the cloaca is abnormally formed. Comparison of the corresponding brightfield images (N–P) suggests the cloaca in the *wdpcp* morphant may be obstructed. The arrowhead denotes the obstructed cloaca, which was seen in 37% of the *wdpcp* morphants (*n* = 208).
(JPG)

Figure S4 **Rescue of** ***wdpcp*** **morpholino (MO)-induced phenotype.** (A) Representative images of 48 hpf embryos injected at one-cell stage with 10 ng of scrambled control MO. (B) Representative images of 48 hpf embryos injected with 10 ng of *wdpcp* MO at one-cell stage showing pericardial edema (black arrows) and a curved tail. (C) Representative images of 48 hpf embryos injected at one-cell stage with 200 pg synthetic mouse *wdpcp mRNA*. (D) Representative images of 48 hpf embryos co-injected at one-cell stage with 10 ng of *wdpcp* MO and 200 pg synthetic mouse *wdpcp mRNA* showing rescue of morphant phenotype. (E, F) Morphant phenotypes (normal, mild, and severe) obtained in the experiments examining *wdpcp* mRNA rescue of *wdpcp* MO-injected embryos are summarized in the graph shown in (F) and the table in (G).
(JPG)

Figure S5 **Production and phenotype of** ***Wdpcp*** **knockout mice.** (A) Schema of the *Wdpcp* targeted mouse allele generated by homologous recombination containing an FRT-flanked PGKneo cassette bracketed with two loxp sites that would allow the deletion of exon 5 to generate a *Wdpcp* knockout allele. (B–E) Newborn homozygous *Wdpcp* knockout mouse exhibited craniofacial defects (B), congenital heart defects (pulmonary atresia) (C), limb polydactyly (D), and duplex kidney (arrows in E), phenotypes identical to those seen in the *Wdpcp^Cys40^* mutants. Scales bars, 200 µm in (C–E).
(JPG)

Figure S6 **Shh signaling is compromised in** ***Wdpcp^Cys40^*** **mutants.** (A) In *Wdpcp^Cys40^* mutant embryos (E10.5 dpc), caudal neural tube (between the forelimb and hindlimb) showed diminished FoxA2 in the ventral floorplate, and expansion of Olig2 and Pax6 to the ventral most position. (B) Smoothened agonist SAG treatment upregulated *Gli1* expression in wild-type MEFs by 20-fold, while *Wdpcp^Cys40^* mutant MEFs were not responsive to SAG stimulation. (C) Western blot of E10.5 whole embryo extract showed *Wdpcp^Cys40^* homozygous mutants had higher Gli3-FL/Gli3-R ratio compared to wild-type controls, indicating impaired Gli3 processing. (D) Western blot of E10.5 neural tube extract showed elevated Gli2-FL level in *Wdpcp^Cys40^* mutant. Scales are the same for images in (A), and the scale bar is 50 µm.
(JPG)

Movie S1 **Nodal cilia show normal motility in** ***Wdpcp^Cys40^*** **mutant embryos.** DIC (Differential Interference Contrast) imaging showed similar nodal cilia motility in wild-type (left) and homozygous *Wdpcp^Cys40^* mutant (right) E8.0 embryos. Scale bar, 10 µm.
(MOV)

Movie S2 **3D reconstruction showing Wdpcp and Sept2 in ring-like structure.** The confocal image stack used to generate the images shown in Figure 2I–L was reconstructed in three dimension (3D) to show ring-like structure comprising Wdpcp (red) with Sept2 (green).
(MOV)

Movie S3 **Motile cilia in the** ***Wdpcp^Cys40^*** **mutant tracheal respiratory epithelium.** The ciliated tracheal epithelia of a near-term *Wdpcp^Cys40^* mutant fetus showed normal coordinated ciliary motion.
(MOV)

Movie S4 **Motile cilia in the zebrafish pronephric duct showed weak and uncoordinated beat after** ***wdpcp*** **morpholino knockdown.** The cilia in the pronephric duct showed strong coordinated beat in zebrafish embryo injected with control morpholino, but in *wdpcp* morpholino-injected embryos (bottom five panels), cilia in the pronephric duct showed weak and uncoordinated beat.
(MOV)

Movie S5 **Time-lapse imaging shows decreased membrane ruffling in** ***Wdpcp^Cys40^*** **mutant MEF.** Time-lapse imaging shows dynamic membrane ruffling in wild-type MEF (top panel). In contrast, *Wdpcp^Cys40^* mutant MEF (bottom panel) showed much less membrane ruffling activity, with unusually long thin filopodial extensions that were immotile. Images were captured every 5 s over a 320 s interval.
(MOV)

Table S1 **Primer sequences for cloning and qPCR.** Rows 1 and 2, primers used to clone *Wdpcp* for making FLAG–Cys40 construct. Rows 3–34, primers used for heart outflow tract qPCR.
(DOCX)

## Abbreviations

AA or aa: amino acid
AER: apical ectodermal ridge (limb)
AVSD: atrioventricular septal defects (heart)
BBS: Bardet–Biedl Syndromes
Co-IP: coimmunoprecipitation
DBA: Dolichos biflorus agglutinin
DL: distal early tubule (kidney)
EFIC: episcopic fluorescence image capture
EM: electronic microscopy
ENU: ethylnitrosourea
ES cells: embryonic stem cells
IHC: inner hair cell (cochlea)
KO: knockout
LV: left ventricle (heart)
MA: major axis
MEF: mouse embryonic fibroblast
MFP: membrane fluctuation period
MI: minor axis
MKS: Meckel–Gruber Syndromes
MO: morpholino
NGS: normal goat serum
OFT: outer flow tract (heart)
OHC: outer hair cell (cochlea)
PAtr: pulmonary atresia (heart)
PBT: phosphate buffered saline with Tween-20
PCP: planar cell polarity
PCR: polymerase chain reaction
PD: pronephric duct (kidney)
PST: proximal straight (kidney)
PTA: persistent truncus arteriosus (heart)
Phal: Phalloidin
Ptch1: Patched1
SMA: smooth muscle actin
Sept2: septin 2
Shh: Sonic hedgehog
Smo: Smoothened
TEF: tracheoesophageal fistula
