# Supplementary figures and images for: Wdpcp, a PCP Protein Required for Ciliogenesis, Regulates Directional Cell Migration and Cell Polarity by Direct Modulation of the Actin Cytoskeleton
Source: PLoS Biol. 2013 Nov 26;11(11):e1001720. doi: 10.1371/journal.pbio.1001720 (PMC3841097; doi:10.1371/journal.pbio.1001720)

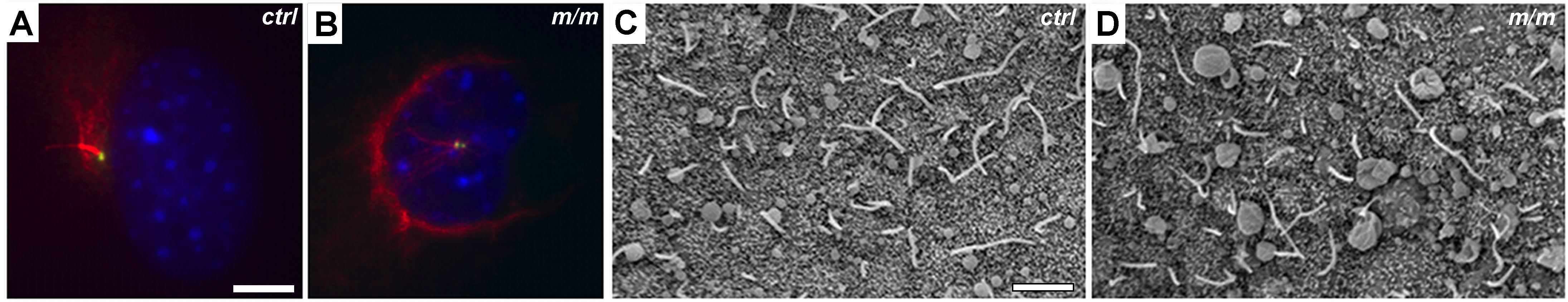

Supplement: Figure S1 — Ciliogenesis defect in WdpcpCys40 mutant. (A, B) Immunostaining of cilium with acetylated α-tubulin, a-tub (red), and γ-tubulin, (g-tub, green) antibodies showing a shorter cilium in WdpcpCys40 mutant MEF (B). Scanning EM images of embryonic nodes of control (C) and mutant (D) embryos at E8.0 showing the node cells are ciliated normally and cilia in mutant embryonic node are of normal shape and length. Scale bars, 2 µm in (A) and (C). Scales are the same in (A, B) and (C, D). (JPG) [file pbio.1001720.s001.jpg]

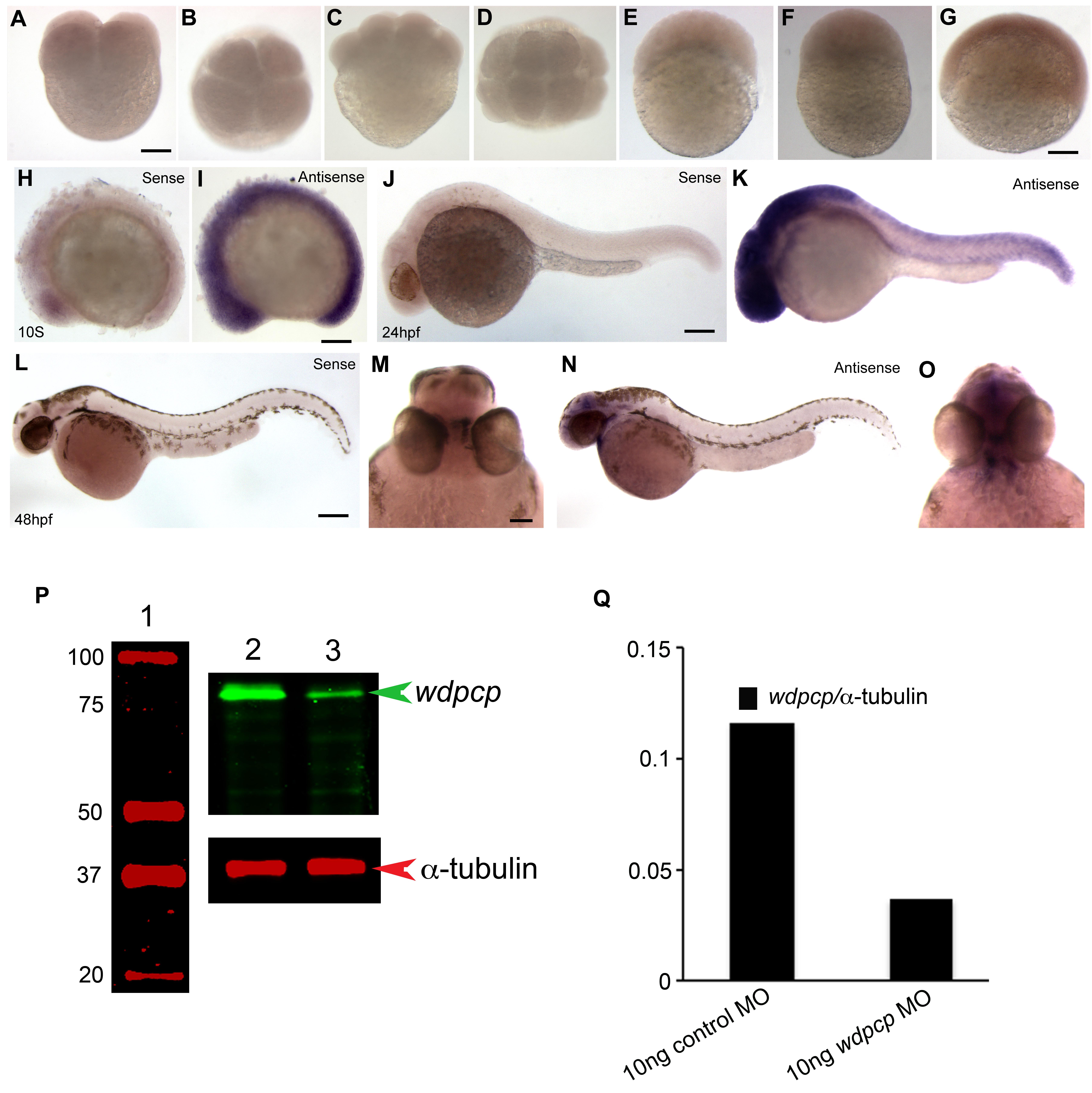

Supplement: Figure S2 — Wdpcp zebrafish in situ hybridization and morphant at 48 h, Western blotting with Wdpcp chicken antibody. (A–O) Embryonic wdpcp mRNA localization (purple) by whole mount in-situ hybridization with wdpcp antisense riboprobe at the four-cell stage (A, B), eight-cell stage (C, D), 1,000-cell stage (E, F), and shield stage (G) showed absence of maternal wdpcp transcripts. Embryonic wdpcp expression is observed at the 10-somite stage (H, I) and at 24 hpf (J, K). At 48 hpf (L–O) wdpcp staining appears less specific, since faint staining is observed with both the sense and antisense probes. (P) Immunoblot using wdpcp antibody (green) with 24 hpf zebrafish embryo lysate showed effective knockdown of wdpcp protein expression. α-Tubulin (red) was used as a sample loading control. Lane 1, protein molecular weight markers; lane 2, lysate from embryos injected with 10 ng control morpholino (MO); and lane 3, lysate from embryos injected with 10 ng wdpcp morpholino. (Q) The ratio of wdpcp (green) to α-tubulin (red) in the immunoblot was quantified using Image studio version 2.0 from LI-COR Biosciences (Lincoln, NE), which showed significant reduction in the wdpcp protein with wdpcp MO knockdown. Scale bars, 200 µm in (A), (G), (I), (J), and (L) and 150 µm in (M). Scales are the same in (A–F), (H, I), (J, K), (L, N), and (M, O). (JPG) [file pbio.1001720.s002.jpg]

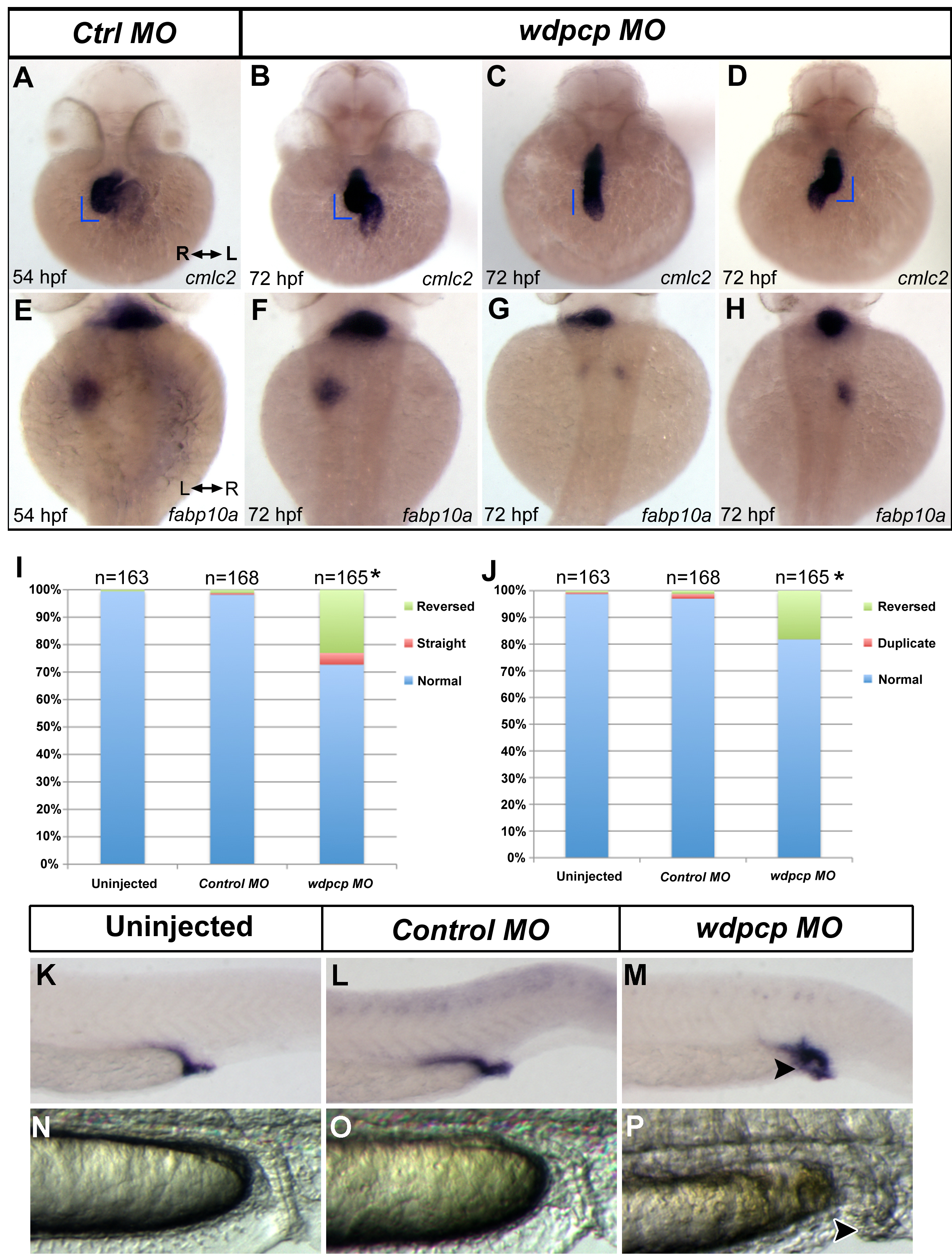

Supplement: Figure S3 — Laterality defects in Wdpcp zebrafish morphants. (A–D) Ventral view of RNA in-situ hybridization staining with cmlc2 probe delineating the heart tube in 54 hpf embryos in wdpcp morphants revealed normal right-sided looping (B), no looping (C), or reversed heart looping (D) orientation. (E–H) Dorsal view of gut orientation as observed with LFABP in-situ hybridization analysis delineating liver position in 54 hpf embryo. Three types of gut orientation were observed: normal left-sided (F), duplicated (G), and right-sided (H). (I, J) Distribution of heart (I) and gut (J) looping orientation in Wdpcp morphants, with asterisk indicating statistically significant differences between control versus wdpcp morphants. (K–P) In-situ hybridization with an evx1 probe on 24 hpf embryos (K–M) delineated the normal cloaca in uninjected (K) and control MO (L) injected embryos, while in the wdpcp morphant (M), the cloaca is abnormally formed. Comparison of the corresponding brightfield images (N–P) suggests the cloaca in the wdpcp morphant may be obstructed. The arrowhead denotes the obstructed cloaca, which was seen in 37% of the wdpcp morphants (n = 208). (JPG) [file pbio.1001720.s003.jpg]

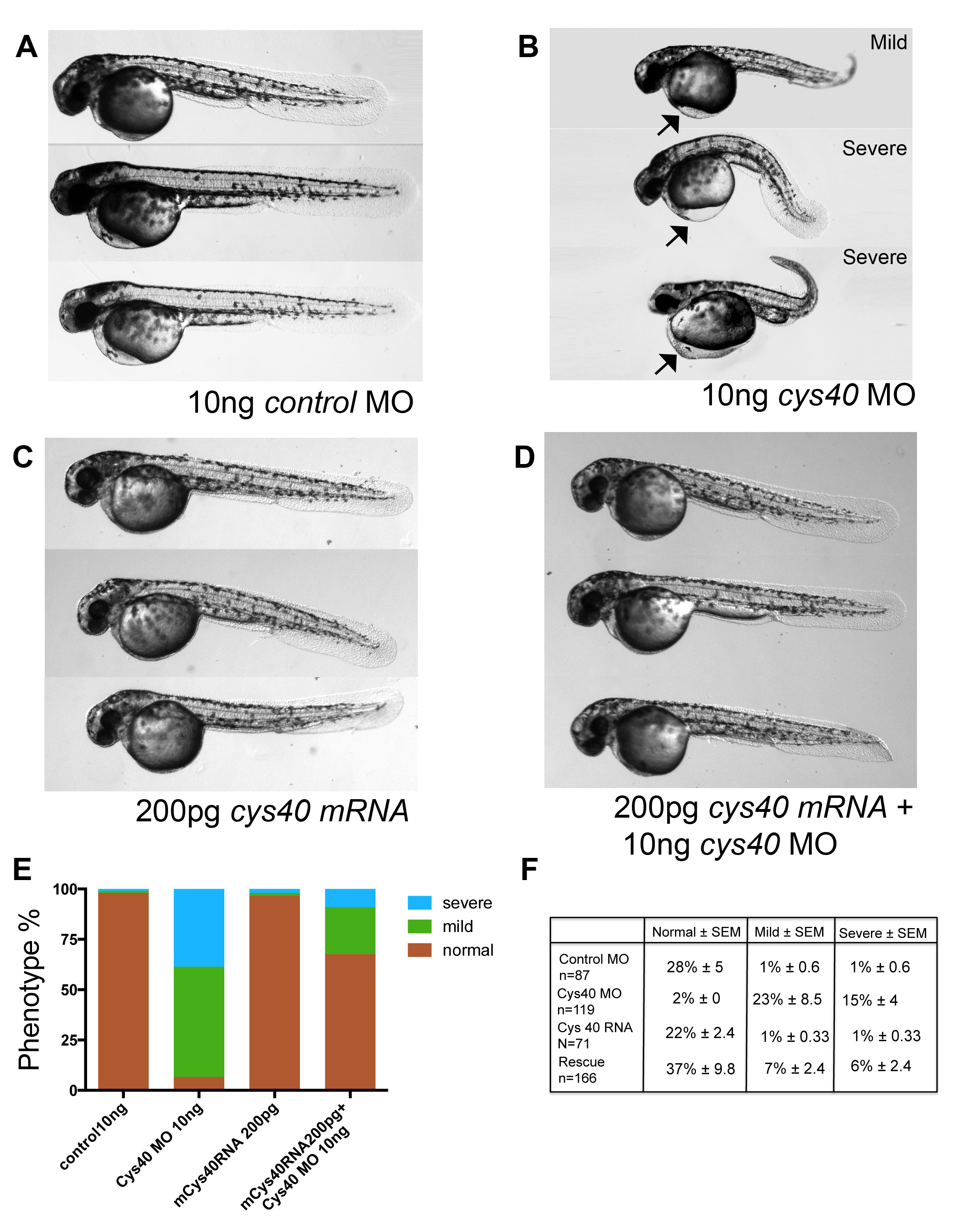

Supplement: Figure S4 — Rescue of wdpcp morpholino (MO)-induced phenotype. (A) Representative images of 48 hpf embryos injected at one-cell stage with 10 ng of scrambled control MO. (B) Representative images of 48 hpf embryos injected with 10 ng of wdpcp MO at one-cell stage showing pericardial edema (black arrows) and a curved tail. (C) Representative images of 48 hpf embryos injected at one-cell stage with 200 pg synthetic mouse wdpcp mRNA. (D) Representative images of 48 hpf embryos co-injected at one-cell stage with 10 ng of wdpcp MO and 200 pg synthetic mouse wdpcp mRNA showing rescue of morphant phenotype. (E, F) Morphant phenotypes (normal, mild, and severe) obtained in the experiments examining wdpcp mRNA rescue of wdpcp MO-injected embryos are summarized in the graph shown in (F) and the table in (G). (JPG) [file pbio.1001720.s004.jpg]

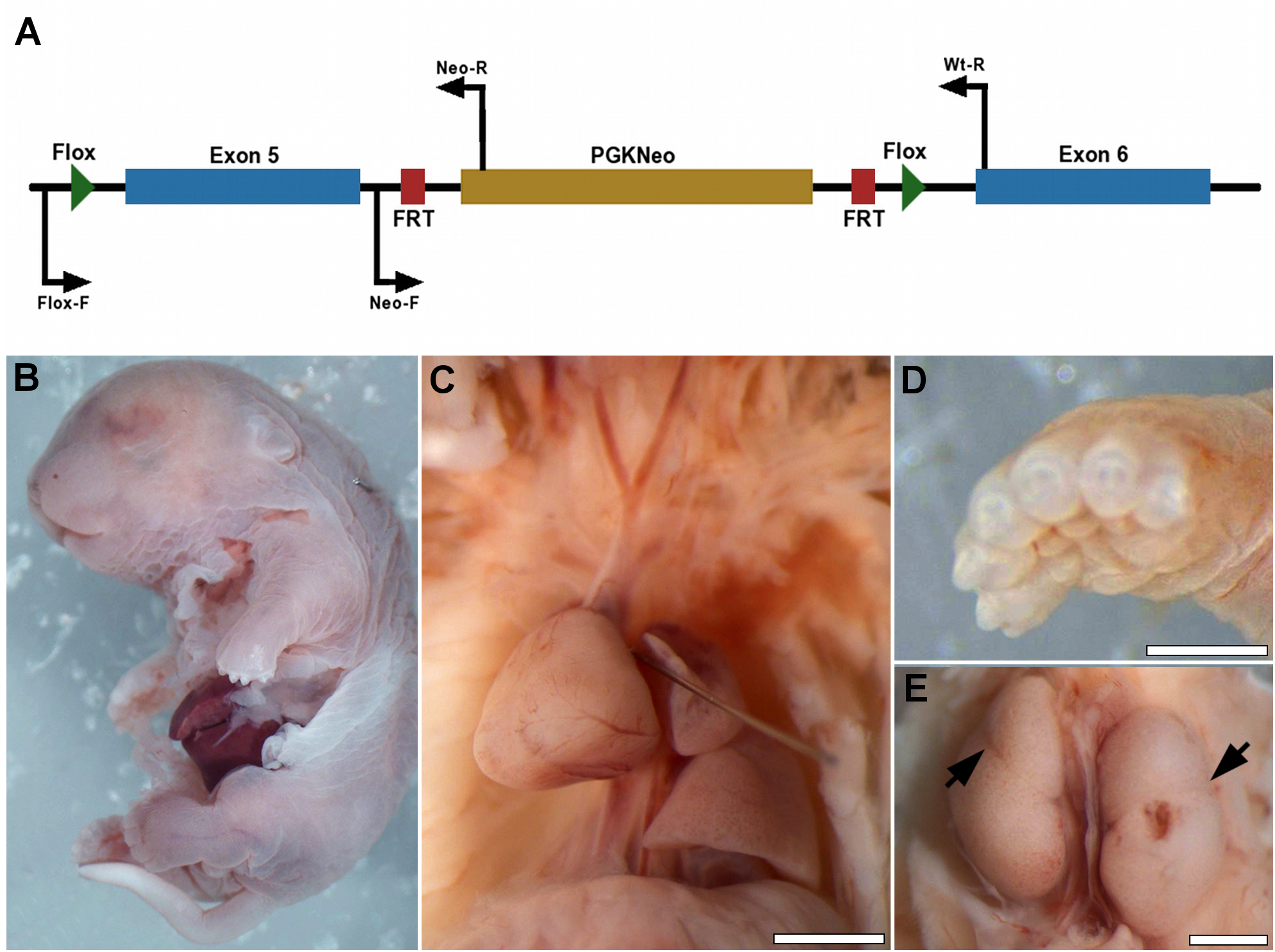

Supplement: Figure S5 — Production and phenotype of Wdpcp knockout mice. (A) Schema of the Wdpcp targeted mouse allele generated by homologous recombination containing an FRT-flanked PGKneo cassette bracketed with two loxp sites that would allow the deletion of exon 5 to generate a Wdpcp knockout allele. (B–E) Newborn homozygous Wdpcp knockout mouse exhibited craniofacial defects (B), congenital heart defects (pulmonary atresia) (C), limb polydactyly (D), and duplex kidney (arrows in E), phenotypes identical to those seen in the WdpcpCys40 mutants. Scales bars, 200 µm in (C–E). (JPG) [file pbio.1001720.s005.jpg]

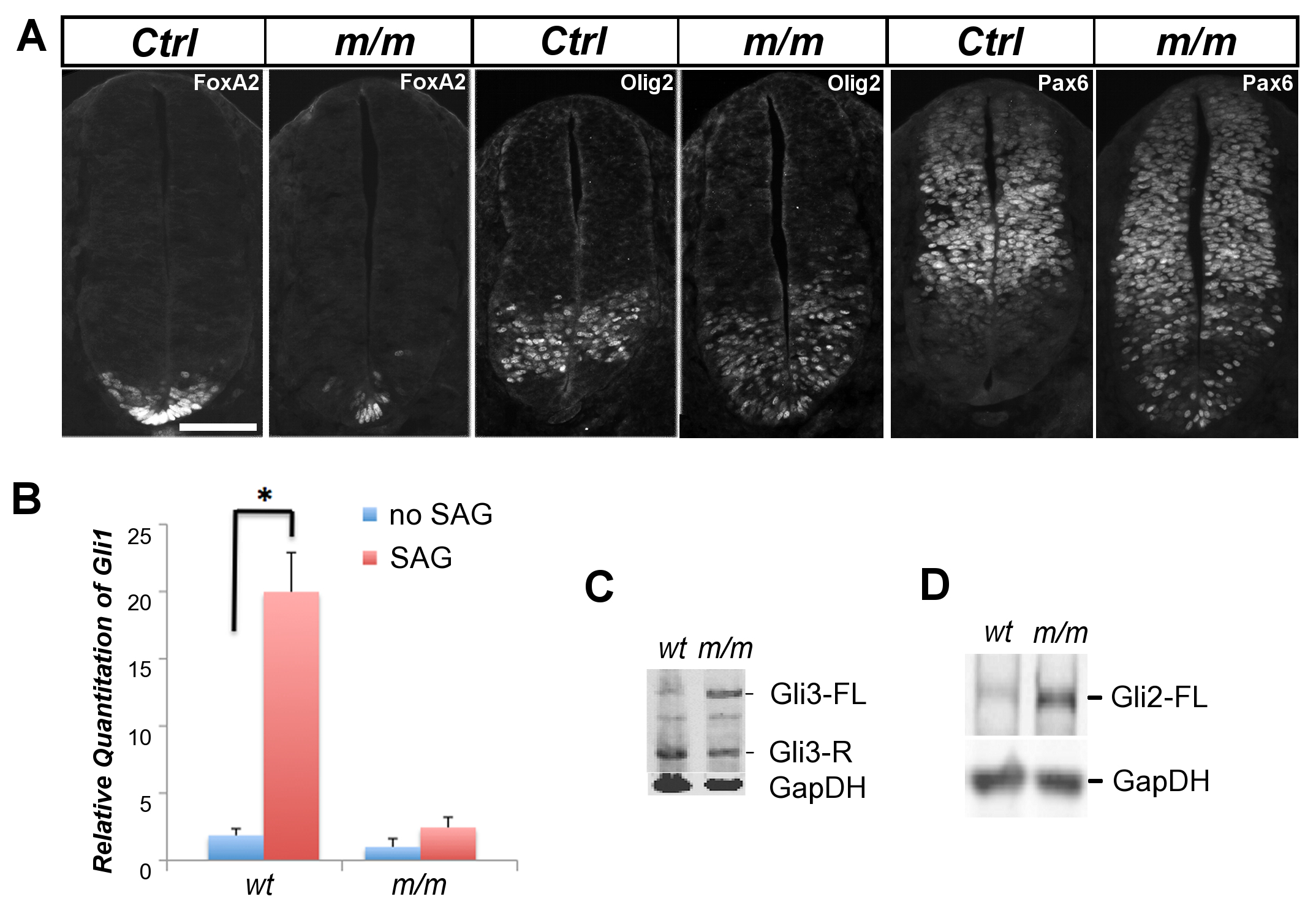

Supplement: Figure S6 — Shh signaling is compromised in WdpcpCys40 mutants. (A) In WdpcpCys40 mutant embryos (E10.5 dpc), caudal neural tube (between the forelimb and hindlimb) showed diminished FoxA2 in the ventral floorplate, and expansion of Olig2 and Pax6 to the ventral most position. (B) Smoothened agonist SAG treatment upregulated Gli1 expression in wild-type MEFs by 20-fold, while WdpcpCys40 mutant MEFs were not responsive to SAG stimulation. (C) Western blot of E10.5 whole embryo extract showed WdpcpCys40 homozygous mutants had higher Gli3-FL/Gli3-R ratio compared to wild-type controls, indicating impaired Gli3 processing. (D) Western blot of E10.5 neural tube extract showed elevated Gli2-FL level in WdpcpCys40 mutant. Scales are the same for images in (A), and the scale bar is 50 µm. (JPG) [file pbio.1001720.s006.jpg]
