# Supplementary material for: Wdpcp, a PCP Protein Required for Ciliogenesis, Regulates Directional Cell Migration and Cell Polarity by Direct Modulation of the Actin Cytoskeleton
Source: PLoS Biol. 2013 Nov 26;11(11):e1001720. doi: 10.1371/journal.pbio.1001720 (PMC3841097; doi:10.1371/journal.pbio.1001720)
Supplement: Table S1 — Primer sequences for cloning and qPCR. Rows 1 and 2, primers used to clone Wdpcp for making FLAG–Cys40 construct. Rows 3–34, primers used for heart outflow tract qPCR. (DOCX) [file pbio.1001720.s012.docx]

|  | **Gene name** | **Primer name** | **Sequence** |
| --- | --- | --- | --- |
| 1 | Wdpcp | MmWD40-5kpn | GGGGTACCATGTCTTTCTGCTTGACTGAACTACACCTG |
| 2 | Wdpcp | MmWD40-3kpn | GGGGTACCTTACACCAAACCAAAGTGAACCATTCTGAG |
| 3 | Axin2 | mAxin2-SG-QRT-F1 | ctgctggtcaggcaggag |
| 4 | Axin2 | mAxin2-SG-QRT-R1 | tgccagtttctttggctctt |
| 5 | Ctnnb1 | mCtnnb1-SG-QRT-F | gcagcagcagtttgtgga |
| 6 | Ctnnb1 | mCtnnb1-SG-QRT-R | tgtggagagctccagtacacc |
| 7 | Dkk1 | mDkk1-SG-QRT-F | ccgggaactactgcaaaaat |
| 8 | Dkk1 | mDkk1-SG-QRT-R | ccaaggttttcaatgatgctt |
| 9 | Dkk2 | mDkk2-SG-QRT-F | ctggtacccgctgcaataat |
| 10 | Dkk2 | mDkk2-SG-QRT-R | catggttgcgatctctatgc |
| 11 | Dkk3 | mDkk3-SG-QRT-F | tcgtgaccagatccagctt |
| 12 | Dkk3 | mDkk3-SG-QRT-R | agccgctgcatgtttgtt |
| 13 | Dvl1 | mDvl1-SG-QRT-F | ccatggaccaggacttcg |
| 14 | Dvl1 | mDvl1-SG-QRT-R | ggcaacttggcattgtcat |
| 15 | Dvl2 | mDvl2-SG-QRT-F | acttcaccctccctcgaaa |
| 16 | Dvl2 | mDvl2-SG-QRT-R | gaggagccagggtaagcag |
| 17 | Dvl3 | mDvl3-SG-QRT-F | caccgtcactctcaacatgg |
| 18 | Dvl3 | mDvl3-SG-QRT-R | catcacctcgctcattgct |
| 19 | Gpr177 | mGpr177-SG-QRT-F | tgtatgcaccatcccataagaa |
| 20 | Gpr177 | mGpr177-SG-QRT-R | gctgtggacacccaggtc |
| 21 | Gsk3b | mGsk3b-SG-QRT-F | ttctacaggacaagcgatttaaga |
| 22 | Gsk3b | mGsk3b-SG-QRT-R | cggactatgttacagtggtctagc |
| 23 | Nkd1 | mNkd1-SG-QRT-F | gacaccaaacccgctgag |
| 24 | Nkd1 | mNkd1-SG-QRT-R | tggctgtcaccctggaac |
| 25 | Nkd2 | mNkd2-SG-QRT-F | tgtggaacatcgctcacg |
| 26 | Nkd2 | mNkd2-SG-QRT-R | ggcccctccttagggtct |
| 27 | Wnt5a | mWnt5a-SG-QRT-F | acgcttcgcttgaattcct |
| 28 | Wnt5a | mWnt5a-SG-QRT-R | cccgggcttaatattccaa |
| 29 | Vangl1 | mVangl1-SG-QRT-F | gtgaagaagcggagagcaag |
| 30 | Vangl1 | mVangl1-SG-QRT-R | gggtccatcacctctccag |
| 31 | Vangl2 | mVangl2-SG-QRT-F | ccagccgcttctacaatgtc |
| 32 | Vangl2 | mVangl2-SG-QRT-R | tctccaggatccacactgc |
| 33 | b-actin | mActb-SG-QRT-F | ctaaggccaaccgtgaaaag |
| 34 | b-actin | mActb-SG-QRT-R | accagaggcatacagggaca |

Table S1
